# An integrated experimental–computational framework for elasto-plastic characterization and spatial mapping of canine bone properties

**DOI:** 10.1371/journal.pone.0361283

**Published:** 2026-10-23

**Authors:** Behzad Amirzade, Tareq Zobaer, Janis Lapsley, Do-Gyoon Kim, Laura E. Selmic, Ali Nassiri

**Affiliations:** 1 Department of Integrated Systems Engineering, College of Engineering, The Ohio State University, Columbus, Ohio, United States of America; 2 Simulation Innovation and Modeling Center, The Ohio State University, Columbus, Ohio, United States of America; 3 Department of Veterinary Clinical Sciences, College of Veterinary Medicine, The Ohio State University, Columbus, Ohio, United States of America; 4 Division of Orthodontics, College of Dentistry, The Ohio State University, Columbus, Ohio, United States of America; University of Padova: Universita degli Studi di Padova, ITALY

## Abstract

Bone is a hierarchical and heterogeneous bio-composite material, with cortical and trabecular regions exhibiting distinct mechanical behaviors across scales. Patient-specific Finite Element Analysis (FEA) derived from medical images is increasingly used in both human and veterinary medicine to predict bone mechanical behavior under various loading conditions, assess fracture risk, and evaluate treatment outcomes. However, due to the complexity associated with measuring full elasto-plastic bone properties across multiple scales and bridging microscale properties to macroscale models, simplified material definitions are often adopted in FEA simulations. Such simplifications may produce unrealistic mechanical responses and fail to capture permanent microscopic and macroscopic deformations that occur when stresses exceed the bone yield point, particularly in implant design applications. This study presents an integrated experimental–computational framework for generating continuum material maps as a function of density from tissue-scale characterization of the canine femur. High-depth spherical nanoindentation combined with Genetic Algorithm (GA)-based inverse optimization was conducted to extract mechanically consistent elasto-plastic properties of cortical and trabecular bone from load–displacement responses influenced by post-yield contact uncertainties. Trabecular tissue-scale properties were then assigned to Representative Volume Elements (RVEs) reconstructed from micro-Quantitative Computed Tomography (μQCT) data and homogenized to account for architecture-dependent effective behavior. Finally, optimized cortical and homogenized trabecular properties were correlated with density to establish compartment-specific material mapping relationships for whole-bone FEA simulations. The optimized responses showed good agreement with the experimental indentation measurements, supporting the capability of the proposed framework to generate mechanically informed material definitions for patient-specific bone FEA simulations. Overall, the proposed framework provides a pathway for translating localized experimental measurements into heterogeneous elasto-plastic material inputs for whole-bone FEA without explicitly resolving trabecular microarchitecture.

## 1. Introduction

Bone is a hierarchical and heterogeneous material composed of organic and mineral phases, with cortical and trabecular compartments showing distinct density, architecture, and mechanical behavior [1,2]. Accurate characterization of these compartments is essential for developing reliable constitutive models for Finite Element Analysis (FEA), particularly for predicting bone deformation and fracture. This is clinically important because reconstruction from medical images is increasingly used to generate patient-specific bone models for FEA, supporting fracture-risk assessment, treatment evaluation and planning, and rapid clinical triage.

Recent studies have utilized Computed Tomography (CT) and micro-Quantitative Computed Tomography (µQCT) images to generate subject-specific FEA models of bone for estimating damage and fracture risk [3–6]. In these models, heterogeneous material properties are commonly assigned using relationships between Hounsfield Unit (HU), bone mass density, and elastic modulus, especially for clinical CT-derived FEA. Other studies have also used HU-dependent material mapping to represent spatial variation in bone stiffness and improve anatomical realism in FEA simulations [6–8]. However, the accuracy of these models strongly depends on the selected modulus–density relationship. Pervez et al. [9] showed that different modulus–density equations can substantially affect FEA predictions, particularly when the relationships are not bone-specific or do not distinguish between cortical and trabecular compartments. In addition, many bone FEA studies rely on empirical modulus–density equations from the literature rather than relationships derived from the actual local bone architecture of the modeled specimen. Therefore, current knowledge supports the importance of heterogeneous CT-based material mapping, but also shows that stronger links are needed between experimentally measured tissue properties and compartment-specific material assignment for high-fidelity whole-bone FEA.

Mechanical properties of bone can be measured at different scales, from macroscale compression, tension or bending tests that capture bulk response to nanoindentation methods that characterize local tissue-level elastic and plastic behavior [10–12]. Although nanoindentation characterization of human bone has been widely reported under both wet and dry conditions [13–16], site-specific elasto-plastic properties of canine bone, especially beyond elastic modulus and hardness, remain limited. Canine bone studies have mainly focused on limited regions such as the femoral head, distal metaphysis, or cortical diaphysis [17–22]. This limitation is particularly relevant in veterinary applications, where accurate material properties are needed to assess bone strength and fracture risk in conditions such as osteosarcoma [23,24]. Canine bone is also widely used in musculoskeletal research; however, species-specific differences remain important, as canine trabecular bone has been reported to exhibit lower elastic modulus and fatigue strength than human trabecular bone [25,26].

Many previous bone nanoindentation studies have used Berkovich tips, mainly on human or other non-canine species, to obtain elastic modulus rather than full stress–strain or post-yield behavior. Spherical indenters provide a larger initial elastic regime and a smoother, progressively evolving contact stress field, allowing the mechanical response to be followed from elasticity through yielding and into post-yield behavior [27]. Their rounded geometry can also reduce stress concentrations and the likelihood of premature cracking or sudden fracture during indentation of brittle or fracture-sensitive materials. Thus, limited information is currently available on site-specific elasto-plastic properties of canine long bone obtained through spherical nanoindentation, although such properties are important for accurate FEA modeling.

Despite the wide use of Hertzian and Oliver–Pharr indentation analysis [28,29], converting spherical nanoindentation load–displacement data into reliable stress–strain curves remains challenging for biological tissues. Bone violates many of the assumptions underlying these analytical models because it is heterogeneous, anisotropic, porous, and may exhibit non-fully elastic unloading behavior [30–33]. In addition, the complex and nonuniform elastic–plastic stress field beneath a spherical indenter makes direct conversion of indentation data into representative stress–strain curves non-trivial [34,35]. Therefore, a numerical analysis framework is required to regularize the interpretation of nanoindentation data by matching the experimental load–displacement response with FEA predictions and extracting more reliable elasto-plastic material properties.

Moreover, accurate material assignment remains a major challenge in FEA models derived from CT images. In patient-specific whole-bone simulations, the image resolution is usually insufficient to resolve the individual trabeculae matrix and local microstructural connectivity of trabecular bone. Guha et al. [36] demonstrated that continuum FEA implemented directly on a CT voxel grid can estimate trabecular bone modulus by assigning density-based material properties to CT voxels, thereby avoiding explicit segmentation of trabecular architecture. However, their approach required an optimized mesh size of 100 µm. Although this resolution is practical for a small trabecular Region of Interest (ROI), its application to whole long-bone models (e.g., with lengths of 200–300 mm) would result in a prohibitively large number of elements on the order of million elements and substantially increased computational cost, limiting its scalability for patient-specific analysis of entire bones. Therefore, although high-resolution continuum FEA constructed directly from CT voxels is useful for localized trabecular modulus estimation, directly applying such fine voxel or mesh resolution to patient-specific whole-bone fracture simulations remains impractical. This creates a need for a micro-to-continuum material mapping approach that homogenizes microscale properties and microstructural effects into mechanically meaningful continuum-level properties suitable for clinical-scale canine long-bone FEA.

This study aims to propose a systematic pathway that: I) experimentally characterize the microscale elasto-plastic response of both trabecular and cortical regions of canine long bone, specifically the femur of a large-breed dog, using spherical nanoindentation across different anatomical sites; II) develop a Genetic Algorithm (GA)-based inverse optimization framework to improve the extraction of stress–strain behavior from force–displacement data by linking the experimental response with replicated FEA indentation simulations; and III) translate the optimized microscale properties to the continuum scale through trabecular bone homogenization and the development of compartment-specific modulus–density relationships for canine long bone. Together, these objectives support high-fidelity elasto-plastic material assignment for clinical CT-derived FEA models, where trabecular microstructure cannot be explicitly captured in detail, and provide physically relevant material inputs for fracture-risk assessment and biomechanical simulation. The novelty of this work lies in integrating these experimental and computational steps into a unified framework that translates localized nanoindentation measurements into spatially varying material-property maps for whole-bone FEA, as illustrated in Fig 1.

**Fig 1:**
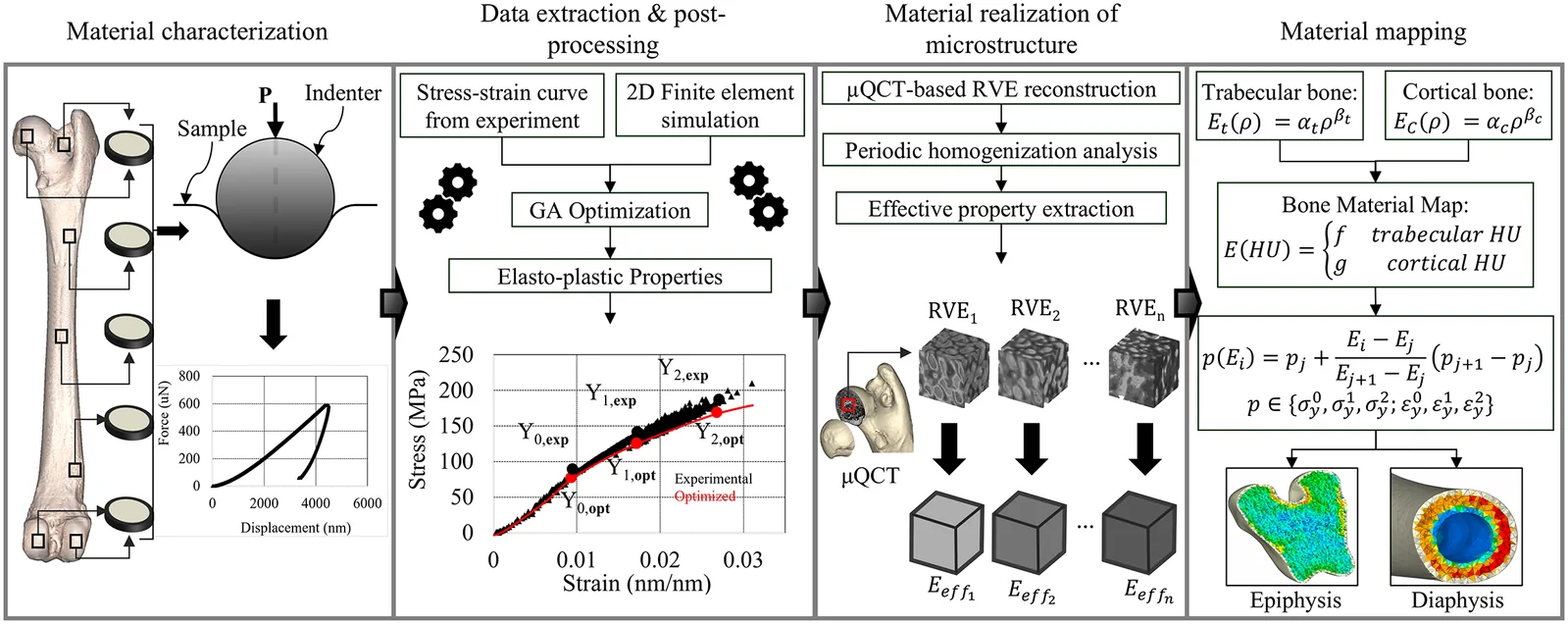
Overview of the proposed integrated experimental-numerical framework. The workflow includes material characterization using spherical nanoindentation; data extraction and post-processing using GA-based inverse identification of elasto-plastic parameters; material realization of microstructure using RVE homogenization; and elasto-plastic material mapping.

The remainder of this paper is organized as follows. First, the nanoindentation experimental setup and testing procedure for microscale characterization of trabecular and cortical bone are described, followed by the extraction of elasto-plastic properties from the nanoindentation results. Next, the material mapping framework is presented, including the homogenization scheme used to obtain effective trabecular properties and the development of modulus–density correlations for canine long bone. The experimental results, numerical optimization outcomes, and material maps are then presented and discussed. Finally, the main conclusions and future implications of the study are summarized.

## 2. Methodology

### 2.1. Experimental setup and procedure

Bone specimens were obtained in dry condition from the femur of a large-breed dog provided by the Department of Veterinary Clinical Sciences at The Ohio State University. Six specimens were sectioned at different locations using a Leco VC-50 precision saw with a diamond cut-off wheel. The sections were cold-mounted in a two-part Leco epoxy resin (Leco Epoxy Resin and Hardener at a 100:14 mass ratio) under vacuum to promote resin infiltration into the porous bone structure and enhance specimen stability during preparation and indentation. Complete epoxy support around the base of each trabecula is important to minimize mechanical artifacts; incomplete embedding may allow a trabecula to deform as a cantilevered beam during indentation, potentially leading to inaccurate measurements of its mechanical properties. After curing for 8–12 hours at room temperature, the mounted samples were ground and polished using a Buehler EcoMet 3000 semi-automatic grinder/polisher. Grinding was performed sequentially with 600, 800, and 1200 grit silicon carbide papers, followed by polishing with 3 µm diamond paste and 0.05 µm colloidal silica. Between each step, the samples were cleaned with water and reagent-grade ethanol and dried to minimize surface contamination and polishing artifacts. These preparation steps were used to progressively reduce surface defects and surface roughness and to improve the consistency and reliability of the nanoindentation measurements, consistent with preparation approaches reported in previous bone nanoindentation studies [37,38]. Representative mounted samples and optical microscopy images of the prepared cross-sections are shown in Fig 2. Microscopic images were acquired at 100 × magnification using an Olympus GX53 inverted optical microscope in brightfield (BF) mode with PAXcam software.

**Fig 2:**
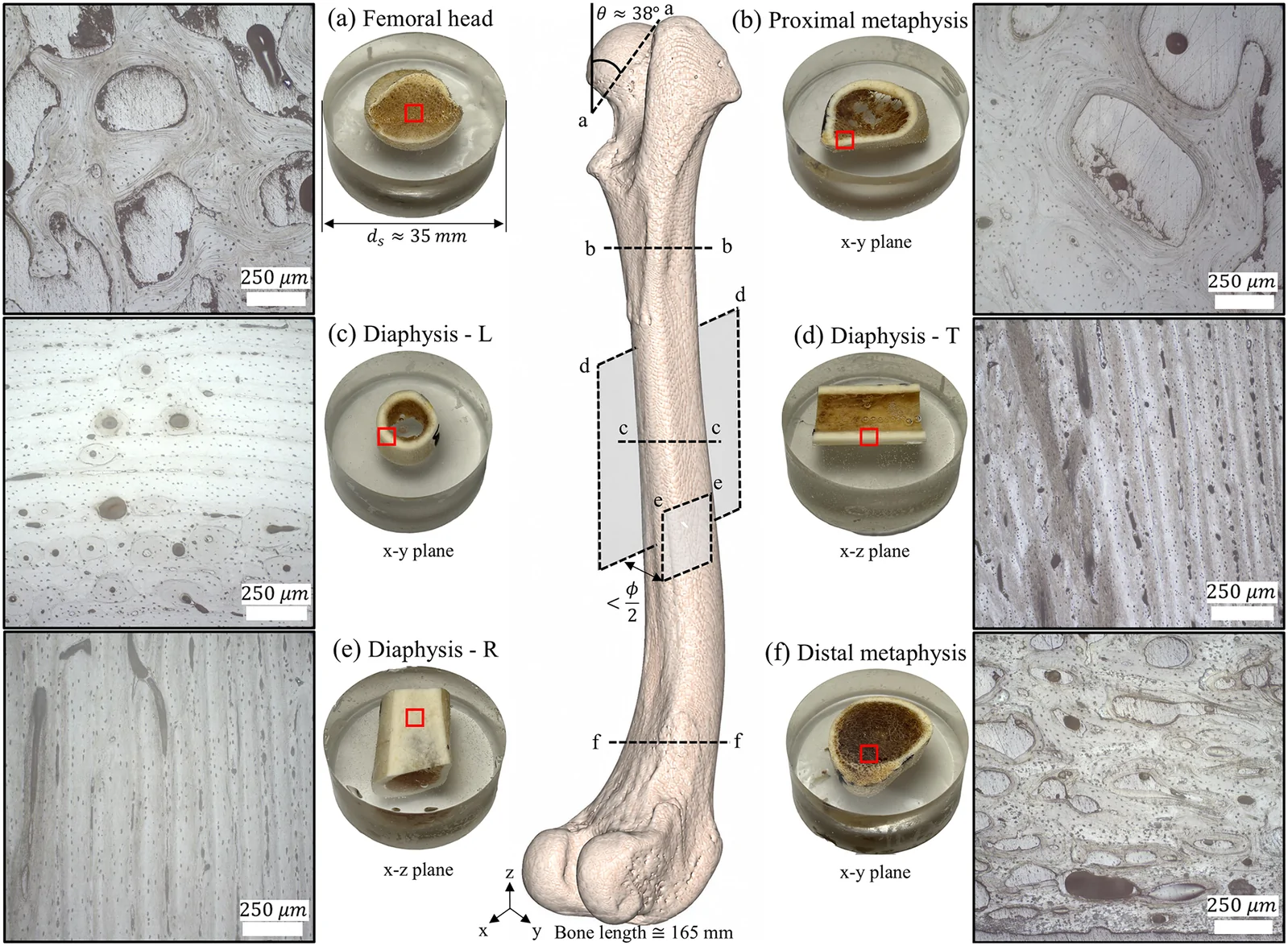
Embedded bone samples in epoxy and the corresponding optical micrographs. Sections a–f were taken from the (a) femoral head, (b) proximal metaphysis, (c) diaphysis – L, (d) diaphysis – T, (e) diaphysis – R, and (f) distal metaphysis. L, T, and R denote longitudinal (parallel to the femoral axis), tangential, and radial directions, respectively. Dashed lines indicate approximate sectioning locations and orientations. Section (a) was obtained at θ ≈ 38°. Red boxes indicate the approximate regions used for optical imaging and nanoindentation. The specimen diameter (d_s_) was approximately 35 mm. Here, ϕ denotes the femoral shaft diameter, and the ϕ/2 offset indicates the near-surface section used to enable radial indentation. Scale bars represent 250 μm.

Spherical nanoindentation tests were conducted using an MTS Nano-XP nanoindenter equipped with a sapphire spherical tip with a diameter of 158 μm, an elastic modulus of 345 GPa, and a Poisson’s ratio of 0.29. The standard Nano-XP system has a maximum normal load capacity of 500 mN, a theoretical force resolution of 50 nN, and a displacement resolution of <0.1 nm [39]. The indentation sites were selected using the built-in optical microscope to ensure that the indents were placed within the target bone regions and away from visible defects or the bone–epoxy interface. To minimize potential local boundary and confinement effects, indentation locations were selected primarily based on surface quality, targeting clean, smooth, and visually intact regions. No additional anatomical or mechanical selection criteria were applied. A custom bone indentation protocol was applied under displacement control, with maximum indentation depths of 4000 nm for trabecular bone and 5000 nm for cortical bone. The protocol was described by Kim et al. [40]. Additional testing parameters included an allowable drift rate of 0.2 nm/s, surface approach distance of 1000 nm, surface approach velocity of 10 nm/s, and settling time of 25 s. Before testing, the indenter tip was calibrated using fused silica [16]. All tests were performed using the continuous stiffness measurement technique. In this technique, a small oscillatory signal is superimposed on the applied load to continuously determine contact stiffness as a function of indentation depth. At each depth, hardness is calculated from the applied load and projected contact area, while the elastic modulus is derived from the measured contact stiffness and contact area, accounting for the elastic properties of the indenter. This approach enables depth-dependent measurement of mechanical properties and is particularly suitable for heterogeneous materials such as bone [41].

#### Ethics statement.

The specimen used in this study was provided by The Ohio State University College of Veterinary Medicine, where standard written owner consent is obtained before treatment as part of the routine clinical consent process.

### 2.2. Elasto-plastic data extraction

Instrumented spherical nanoindentation was used to characterize the local elasto-plastic response of canine bone tissue. The initial elastic response of the spherical nanoindentation load–displacement curve was interpreted using Hertzian contact theory [28], which relates the applied load (*P*), indentation depth (*h*_*t*_), tip radius (R), and reduced elastic modulus (E^*^) under purely elastic contact conditions, as shown in Fig 3A. The reduced modulus of the sample was introduced to account for the material properties of both the indenter and the specimen:

**Fig 3:**
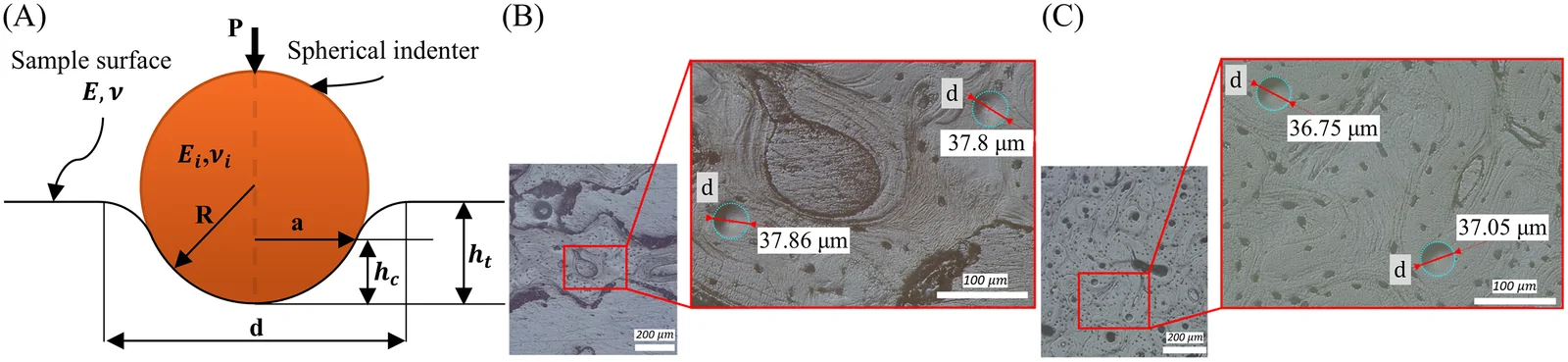
Spherical nanoindentation and representative indentation impressions. (A) Schematic of the spherical indenter in contact with the sample, where a is the projected contact radius at maximum load, h_c_ is the contact depth, h_t_ is the total indentation depth, and d is the residual indentation impression diameter measured after unloading; Representative micrographs of the nanoindentation impressions on (B) trabecular bone, and (C) cortical bone samples. Blue circles indicate the residual indentation impressions.


$$\begin{array}{c} \begin{array}{c} E^{*}=\;{\left[\frac{\text{1}-\nu^{\text{2}}}{E}+\frac{\text{1}-{\nu_{i}}^{\text{2}}}{E_{i}}\right]}^{-\text{1}} \end{array} \end{array}$$ (1)

where *E* and *ν* are the elastic modulus and Poisson’s ratio of the sample, respectively, while *E*_*i*_ = 345 GPa and *ν*_*i*_ = 0.29 are the corresponding properties of the indenter. Once the tip is calibrated, E^*^ can be found using contact depth (*h*_*c*_) and *h*_*t*_, as detailed in S1 Appendix (see Section S1.1). The representative spherical indentation marks on the trabecular and cortical microstructure are also shown in Fig 3B and 3C, respectively.

Spherical indentation differs fundamentally from conical and pyramidal indentation because the spherical tip is geometrically non-self-similar. In self-similar indenters, the contact geometry scales proportionally with penetration depth, whereas in spherical indentation the contact radius, projected contact area, and subsurface stress field evolve continuously and nonlinearly as indentation depth increases [42,43]. This depth-dependent evolution of contact geometry enables the loading response to be associated with a representative indentation strain. Consequently, the spherical indentation load–displacement curve can be transformed into an indentation stress–strain response, providing a more suitable basis for evaluating the elasto-plastic behavior of bone tissue as a semi-brittle material following the approach proposed by Juliano et al. [43]. The indentation stress was calculated as the applied load divided by the projected contact area:


$$\begin{array}{c} \begin{array}{c} \sigma =\frac{\psi P}{\pi a^{\text{2}}}=\text{0}.\text{9}\left(\frac{h_{t}}{h_{c}}-\text{1}\right)\frac{P}{\pi a^{\text{2}}}\; \end{array} \end{array}$$ (2)

where *a* is the projected contact radius and ψ = 0.9 (ht/hc−1) is a depth-dependent correction factor. As indentation progresses, ht/hc changes naturally, allowing the correction factor *ψ* to account for the gradual transition from predominantly elastic contact to elastic-plastic deformation. The factor has been reported to be approximately 0.9 in the purely elastic regime and to decrease as plastic deformation develops [43,44]. After the indentation stress was obtained, the corresponding expression for true strain was calculated using the relationship between indentation stress, elastic modulus, contact geometry, total indentation depth, contact depth, and calibrated projected contact area. The complete derivation and equations used for the indentation strain calculation are provided in Section S1.1 of S1 Appendix.

Once the indentation stress and strain were calculated from the experimental nanoindentation data, the elastic modulus and apparent initial yield point were estimated from the resulting curves. The apparent initial yield point was defined as the onset of deviation from the initial linear response. Following the derivative approach described by Nesbitt et al. [45], this point was identified from the early portion of the curve as the location of maximum slope among the zero crossings of the second derivative. The second derivative was used to identify candidate stationary points in the first derivative, where changes in the evolution of the curve slope occur. This criterion identifies the transition in slope from the initial linear response to the nonlinear regime. In this study, the search region was limited to strains below 1.5% to ensure that the identified point corresponded to the initial elastic-to-nonlinear transition rather than later post-yield behavior. Because the experimentally derived indentation stress–strain curves contained local point-by-point oscillations, particularly after yielding, direct numerical differentiation could become unstable as differentiation amplifies local variations in the data. Therefore, a 12th-degree polynomial was fitted to the data to obtain a smooth representation of the response before calculating the first and second derivatives. The elastic modulus was then determined as the slope of a least-square linear fit over a 0.5% strain interval immediately preceding the identified apparent yield point.

Beyond the elastic regime, direct interpretation of the calculated indentation stress–strain response is intricated by uncertainties in estimating the true contact area. These uncertainties may result from plastic deformation mechanisms, including pile-up, where material accumulates around the indenter, and sink-in, where the surface depresses around the contact region, as well as experimental artifacts such as deviations from the ideal indenter geometry and local surface roughness. Therefore, an inverse numerical framework was employed to mitigate the influence of these post-yield uncertainties and obtain more stable and physically representative plasticity parameters. In this approach, the elasto-plastic properties of the bone samples were estimated using a GA-based inverse optimization framework coupled with FEA. Nanoindentation simulations were performed in ABAQUS [46], while the optimization routine was implemented using an in-house MATLAB [47] code.

For FEA, the bone was modeled as an isotropic elastic material, defined by Young’s modulus and Poisson’s ratio, and isotropic hardening characterized by three data points for yield stress and their corresponding strains. The plasticity model follows classical isotropic (von Mises) plasticity with isotropic hardening. This formulation does not account for the influence of hydrostatic stress on yielding and it was adopted here for the computational tractability within the inverse-identification framework. The effect of Poisson’s ratio on the extracted modulus was not the focus of this study. Therefore, an average value of *ν* = 0.3 was used for bone tissue, consistent with Asgari et al. [48], who reported that varying *ν* from 0.2 to 0.45 changed the estimated modulus by 18.2% for cortical bone and 19.5% for trabecular bone. Frictionless surface-to-surface contact with small sliding was prescribed in the tangential direction between the indenter and the sample, while hard contact was defined in the normal direction to avoid penetration and allow contact pressure to develop [33]. The indenter motion was controlled through a Reference Point (RP), to which the prescribed vertical displacement was applied. The bottom boundary of the bone domain was constrained using an ENCASTRE boundary condition. The indentation process was simulated using a 2D axisymmetric model, with the spherical indenter represented as a rigid surface because its stiffness is substantially higher than that of the bone, making indenter deformation negligible. The bone domain was discretized using 2601 CAX4R four-node axisymmetric quadrilateral elements with reduced integration. A mesh sensitivity analysis was performed by reducing the mesh size until the reaction force recorded at the RP converged to a stable value for a representative test case. In addition, local mesh refinement was applied in the contact region to improve the accuracy of the indentation response. The mesh was spatially graded, with an element edge length of approximately 1 μm in the refined contact region and up to around 10 μm in the coarser regions away from the indentation site. The loading phase of the FEA nanoindentation test was simulated by incrementally applying the experimental maximum penetration depth under static, nonlinear conditions, with reaction forces at the RP extracted from the ABAQUS output. Fig 4A shows the static axisymmetric FEA setup used to simulate the spherical nanoindentation tests.

**Fig 4:**
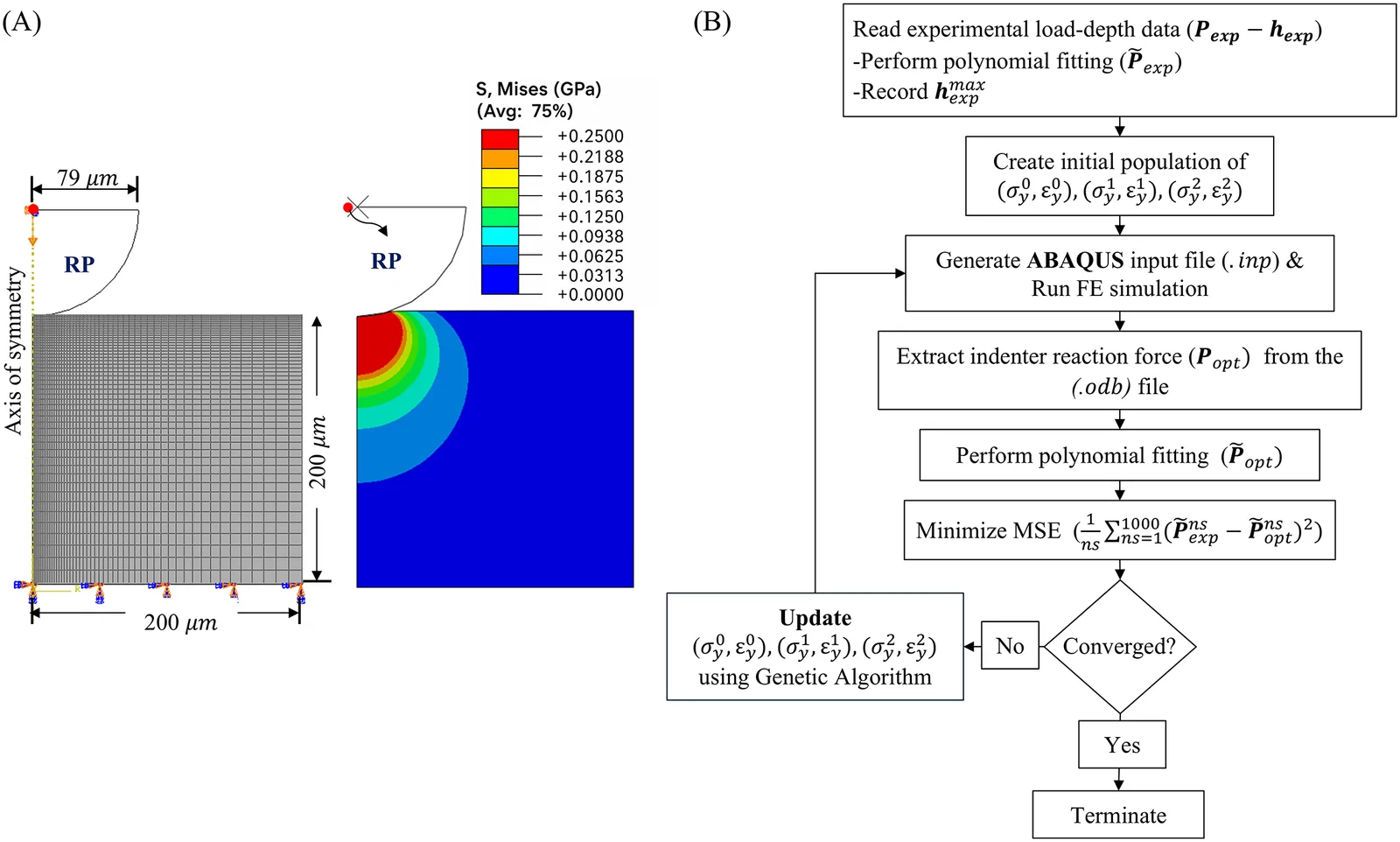
FEA simulation and GA-based inverse optimization framework for spherical nanoindentation. (A) FEA simulation setup replicating the nanoindentation experiment, showing the discretized geometry with refined mesh near the contact region and the resulting post-indentation stress contour. Contour plot of von Mises stress, consistent with the formulation adopted in the constitutive model, is reported for visualization of the simulated stress field only. (B) Flowchart of the inverse optimization framework used to determine elasto-plastic material properties from nanoindentation data. The optimized FEA load (P_opt_) is obtained from the reaction force at the RP for the prescribed indentation displacement and is compared with the experimental load–depth response during optimization.

The primary output extracted from each FEA simulation was the reaction force at the RP as a function of the prescribed indentation depth. This simulated load–depth response was used in the inverse optimization to identify the elasto-plastic material parameters by comparison with the corresponding experimental response. Further FEA implementation details are provided in S1 Appendix (Section S1.2).

The plastic response was defined using three yield stress–strain control points:


$$\begin{array}{c} \begin{array}{c} 𝐱=\left[\sigma_{y}^{\text{0}},\varepsilon_{y}^{\text{0}},\sigma_{y}^{\text{1}},\varepsilon_{y}^{\text{1}},\sigma_{y}^{\text{2}},\varepsilon_{y}^{\text{2}}\right] \end{array} \end{array}$$ (3)

where (*σ*_*y*_^*0*^, *ε*_*y*_^*0*^) represents the initial yield point, (*σ*_*y*_^*1*^, *ε*_*y*_^*1*^) describes an intermediate control point introduced to represent the evolution of the post-yield hardening response, and (*σ*_*y*_^*2*^, *ε*_*y*_^*2*^) is the terminal point within the analyzed strain range. The intermediate point provides flexibility in describing nonlinear hardening rather than representing a separate physical yielding event. These three points define the piecewise isotropic hardening behavior in ABAQUS, where $\varepsilon_{y}^{i}\;(i\;=\;\text{0},\;\text{1},\;\text{2})$ denotes the corresponding total uniaxial strain. These parameters served as optimization design variables. The GA iteratively updated these parameters by minimizing the difference between the experimental load–depth response obtained from nanoindentation and the corresponding FEA-predicted response, allowing the optimized elasto-plastic material properties to be identified. This framework is illustrated in Fig 4B.

To ensure a physically realistic elasto-plastic response, the optimized stress–strain points were constrained to follow a monotonic hardening behavior:


$$\begin{array}{c} \begin{array}{c} \begin{array}{c} \varepsilon_{y}^{\text{0}}\leq \varepsilon_{y}^{\text{1}}\leq \varepsilon_{y}^{\text{2}}\\ \sigma_{y}^{\text{0}}\leq \sigma_{y}^{\text{1}}\leq \sigma_{y}^{\text{2}} \end{array}\; \end{array} \end{array}$$ (4)

These constraints are informed by the observed behavior of the nanoindentation stress–strain curve, which demonstrates a positive correlation between stress and strain. The terminal strain was bounded at 3%, and all simulations remained within the calibrated hardening range, avoiding extrapolation beyond the final defined point. The inverse optimization problem was formulated by minimizing the Mean Squared Error (MSE) between the experimental and FEA-predicted load–depth curves:


$$\begin{array}{c} \begin{array}{c} {\underset{\;𝐱}{\text{min}}\;J\left(𝐱\right)=\;\frac{\text{1}}{N_{s}}\sum_{i=\text{1}}^{N_{s}}\left[{\tilde{P}}_{exp}\left(h_{i}\right)-{\tilde{P}}_{opt}\left(h_{i},𝐱\right)\right]}^{\text{2}}\; \end{array} \end{array}$$ (5)

where *P̃*_*exp*_ and *P̃*_*opt*_ are the experimental and simulated indentation loads obtained from the 12^th^-degree polynomial fit at indentation depth *h*_*i*_, respectively, and *N*_*s*_ *= 1000* is the number of evenly spaced sample points between 0 and *h*_*exp*_^*max*^, used for curve comparison. The polynomial fitting provided a smooth representation of both responses and enabled direct comparison at identical penetration depths. This becomes important for evaluating the objective function of the optimization problem. A polynomial-order sensitivity assessment is provided in Section S1.1 of S1 Appendix. The GA iteratively updated the material parameters until either the maximum of 15 generations was reached or the best fitness value showed no further improvement for five consecutive generations. This stopping strategy was selected to balance convergence accuracy with computational cost, since each fitness evaluation required a separate FEA simulation. Since GA is stochastic in nature, no gradient information is needed here. This approach is advantageous as deriving gradient expressions for such a highly non-convex optimization problem would be exceedingly complex. To assess optimization repeatability, a representative central-diaphysis cortical test in the tangential direction was independently optimized three times using different initial populations. Details of the GA implementation and repeatability assessment are provided in S1 Appendix (see Section S1.2).

### 2.3. Micro-to-continuum scale material mapping

To bridge tissue-scale mechanical properties with continuum-level material inputs for conventional FEA, the optimized material parameters were incorporated into a homogenization scheme and material mapping framework. The localized nanoindentation measurements were not directly extrapolated throughout the bone; rather, they were used as region-specific tissue properties and combined with regional μQCT-derived architectural and density information to establish compartment-specific continuum relationships. Because cortical bone has a relatively dense microstructure compared with trabecular bone, its continuum-level mechanical response was assumed to be closely related to its tissue-level properties. Hence, the optimized microscale cortical bone properties were considered representative of the continuum-scale (i.e., macroscale) cortical material response in this study. In contrast, trabecular bone has a highly porous and architecturally complex structure, making a direct tissue-to-continuum equivalence inappropriate. Therefore, two Representative Volume Elements (RVEs) were randomly extracted from μQCT images of each ROI of the canine femur to capture region-specific trabecular architecture and derive effective elastic moduli through homogenization. The μQCT images were acquired at a spatial resolution of 20 μm, and cubic RVEs with a side length of 2 mm were chosen to provide a continuum-scale representation of the local trabecular microstructure. Although the RVEs were randomly selected, they were chosen to capture the repeating microstructural patterns characteristic of trabecular bone within each ROI. The selections aimed to reflect trabecular structures comparable to those examined by nanoindentation, while also incorporating additional regional architectures identified in the μQCT images. Thus, the RVEs were not necessarily spatially coincident with the nanoindentation sites. Nanoindentation provided tissue-scale properties, whereas the RVEs captured architecture-dependent effective behavior. Fig 5A illustrates the selected ROIs and their corresponding RVEs across different anatomical locations of the canine femur.

**Fig 5:**
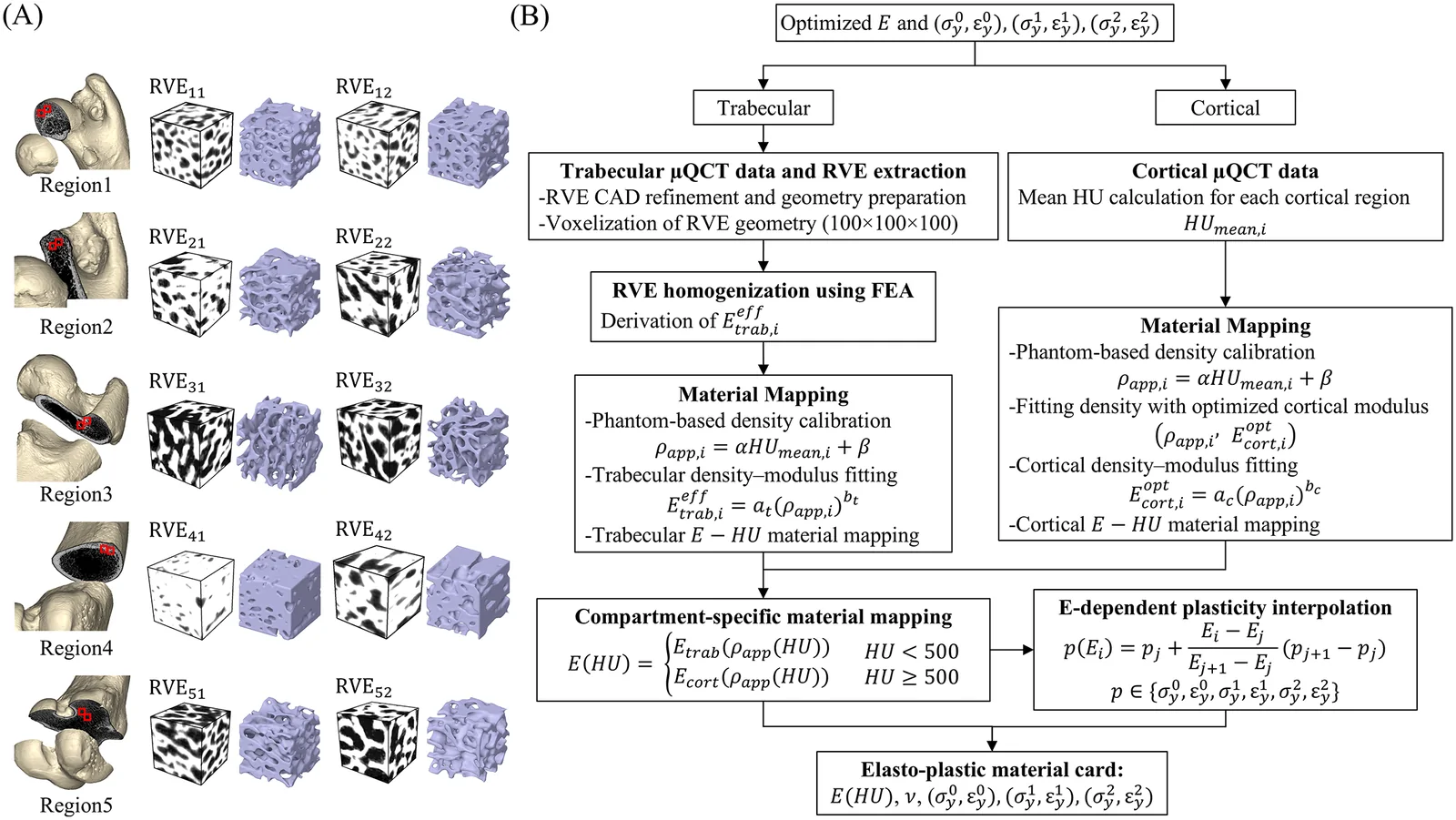
Micro-to-continuum scale material mapping framework. (A) RVEs extracted from femoral head (RVE_11_ and RVE_12_), femoral neck (RVE_21_ and RVE_22_), greater trochanter (RVE_31_ and RVE_32_), distal metaphysis (RVE_41_ and RVE_42_), and distal condyle (RVE_51_ and RVE_52_), where the first index denotes the anatomical region and the second index distinguishes the two RVEs extracted from that region; and (B) workflow for deriving compartment-specific E(HU) relationships and spatially consistent elasto-plastic material properties for FEA.

Each RVE was voxelized using a polygon mesh voxelization algorithm and discretized into a regular finite element unit cell [49]. Six independent unit strain cases were applied to each RVE, including three normal and three shear loading modes, to calculate the homogenized stiffness tensor and the corresponding effective elastic properties. Although the solid bone tissue was assumed to be locally isotropic, the homogenized RVE response was not constrained to isotropy. The six independent loading cases allowed the full homogenized stiffness tensor to be determined, thereby capturing the direction-dependent elastic response of the trabecular architecture. The FEA approximation of the homogenized stiffness tensor for a unit cell with a volume of *|Y|* is given by:


$$\begin{array}{c} \begin{array}{c} E_{ijkl}^{H}=\frac{\text{1}}{|Y|}\sum_{e=\text{1}}^{N_{e}}(u_{e}^{A(ij)}{)}^{T}k_{e}u_{e}^{A(kl)}\; \end{array} \end{array}$$ (6)

In this equation, ***N***_*e*_ is the number of elements, ***k***_*e*_ is the element stiffness matrix, and $u_{e}^{A(ij)}\;$ and $u_{e}^{A(kl)}\;$ are the element displacement fields associated with the applied unit strain fields [50]. Assuming locally isotropic elasticity for the solid bone tissue constituting each voxel, the element stiffness matrix for an element with volume *Ω*_*e*_ is:


$$\begin{array}{c} \begin{array}{c} k_{e}=\int_{\Omega_{e}}B^{T}CB\;d\Omega_{e}\; \end{array} \end{array}$$ (7)

Here ***B*** is the strain–displacement matrix and ***C*** is the constitutive matrix of the solid bone tissue. The constitutive matrix is defined using Lamé parameters:


$$\begin{array}{c} \begin{array}{c} \begin{array}{cc} \lambda =\frac{\nu E}{\left(\text{1}+\nu )(\text{1}-\text{2}\nu \right)}, & \mu =\frac{E}{\text{2}(\text{1}+\nu )} \end{array}\; \end{array} \end{array}$$ (8)

where E and ν are the tissue-scale elastic modulus and Poisson’s ratio, respectively. Note that the Lamé parameters were derived directly from *E* and *ν* and used only to define the element-level constitutive matrix for homogenization.

In this work, the homogenization procedure proposed by Dong et al. [51] for cellular materials was adopted. This approach involved using a voxelization algorithm to project a structure onto a finite element mesh with 3D solid elements. All microstructures were discretized into a unit cell having 100 × 100 × 100 elements. More information on RVE extraction, the homogenization formulation, and the resulting orthotropic effective properties is provided in Section S1.3 of S1 Appendix.

The homogenized elastic properties were used to establish the continuum-scale modulus–density relationship, while the corresponding yield and post-yield parameters were subsequently assigned through the *E*-dependent interpolation procedure. Following homogenization, a density-based material mapping scheme, illustrated in Fig 5B, was developed to assign spatially varying elastic properties to the finite element model. This approach allowed the trabecular compartment to be represented as an equivalent continuum rather than as an explicitly resolved porous architecture, which is not feasible using normal CT resolution. Note that standard phantom calibration was used to convert CT intensity into calibrated density.

For trabecular bone, the average *HU* of each 2 × 2 × 2 mm^3^ RVE from different ROIs was converted to apparent density (*ρ*_*app*_) and paired with its homogenized effective modulus, $E_{trab}^{eff}$, such that each RVE provided an $E_{trab}^{eff}-\rho_{app}$ data point. For cortical bone, the average *HU* of each cortical region was similarly converted to $\rho_{app}$ and paired with the optimized nanoindentation-derived modulus to obtain an $E_{cort}-\rho_{app}$ relationship. Suggested by Helgason et al. [52], a power-law relationship was used to describe both trabecular and cortical bone $E-\rho_{app}\;$ correlation:


$$\begin{array}{c} \begin{array}{c} E\;(\rho_{app})=\left\{ \begin{array}{c} \alpha_{trab}\;\rho_{app}^{\beta_{trab}}\\ \alpha_{cort}\;\rho_{app}^{\beta_{cort}} \end{array}\right.\; \end{array} \end{array}$$ (9)

where *α* and *β* are fitting coefficients determined separately for the trabecular and cortical bone compartments. These compartment-specific $E-\rho_{app}$ relationships can be combined with the phantom-derived $\rho_{app}-HU$ calibration to define $E_{trab}^{eff}(HU)$ and $E_{cort}(HU)$, which enables patient-specific material assignment for both cortical and trabecular regions. Therefore, the final compartment-specific elastic modulus map can be defined as:


$$\begin{array}{c} \begin{array}{c} E(HU)=\left\{ \begin{array}{cc} f_{trab}\left(\rho (HU)\right) & HU<HU_{th}\\ f_{cort}\left(\rho (HU)\right) & HU\geq HU_{th} \end{array}\right.\; \end{array} \end{array}$$ (10)

where *f*_*trab*_ and *f*_*cort*_ are the trabecular and cortical $E-\rho_{app}$ functions, respectively, and *HU*_*th*_ is the threshold separating trabecular- and cortical-dominant regions. This threshold is protocol-dependent and may vary with CT acquisition settings, reconstruction parameters, and calibration procedures; therefore, it should be determined using calibration phantom data when available. Plasticity parameters also can be interpolated as functions of *E(HU)* to assign spatially consistent elasto-plastic properties.

For FEA material assignment, *E(HU)* was used as the intermediate link between CT intensity and the optimized plasticity parameters. Each experimentally calibrated elastic modulus was associated with a corresponding set of plasticity parameters obtained from the inverse optimization, including the initial yield and subsequent hardening stress–strain points. Therefore, when the modulus calculated for a given *HU* interval corresponded to one of the calibrated values, the associated optimized plasticity parameter set was assigned directly. For *HU* intervals producing intermediate modulus values, the corresponding plasticity parameters were obtained by interpolation between the two neighboring calibrated material sets. For a general plasticity parameter p, this can be expressed as:


$$\begin{array}{c} \begin{array}{c} p\left(E_{i}\right)=p_{j}+\frac{E_{i}-E_{j}}{E_{j+\text{1}}-E_{j}}\left(p_{j+\text{1}}-p_{j}\right) \end{array} \end{array}$$ (11)

where *E*_*i*_ is the target elastic modulus, *E*_*j*_ and *E*_*j+1*_ are the neighboring calibrated elastic moduli, and *p*_*j*_ and *p*_*j+1*_ are their corresponding optimized plasticity parameters. This procedure was applied separately to each parameter, *p*∈{σ_y_^0^, ε_y_^0^, σ_y_^1^, ε_y_^1^, σ_y_^2^, ε_y_^2^}, allowing each *HU* interval to be assigned a complete elasto-plastic material card.

This mapping assumes that, within each bone compartment, the optimized plasticity parameters vary smoothly between the experimentally calibrated material states and that their association with the corresponding elastic modulus is representative over the calibrated range. The same constitutive formulation was maintained for all interpolated material sets, and cortical and trabecular properties were treated separately. Thus, *HU* was first used to determine *E* through the established *E(HU)* relationship, and the corresponding plasticity parameters were then assigned through *E*-dependent interpolation between the neighboring calibrated material sets.

## 3. Results

### 3.1. Stress–strain curves

The stress–strain curves derived from nanoindentation are shown in Fig 6. Multiple indentations were performed on each sample to ensure experimental repeatability, and outliers were excluded. The spherical indentation-derived stress–strain curves showed clear site-dependent mechanical behavior, reflecting the heterogeneous nature of the bone tissue. Although variability was observed among indentation points within the same anatomical region, the responses within each site generally followed a similar trend, suggesting that tissue properties were more consistent within a given location than across different anatomical regions.

**Fig 6:**
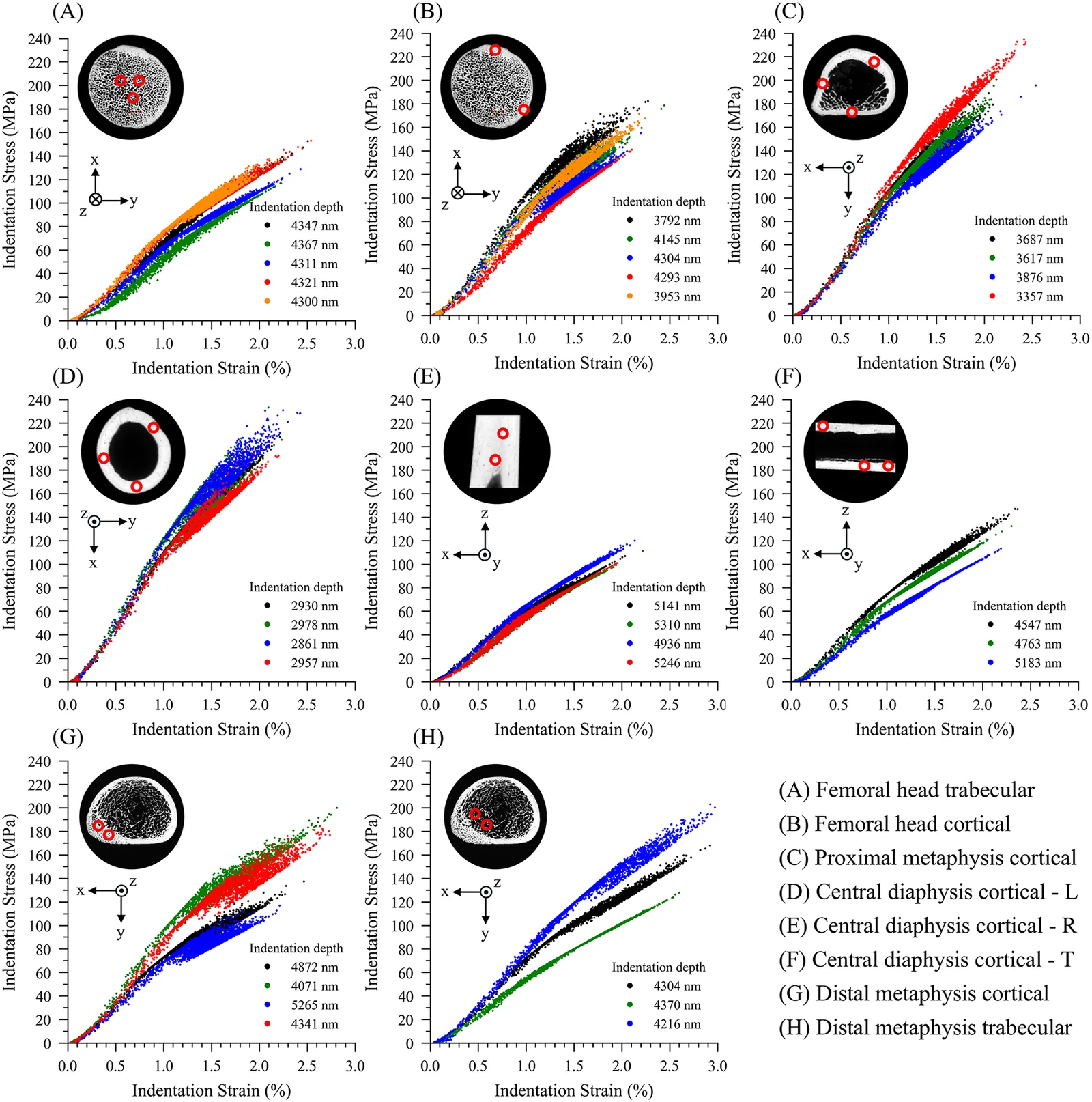
Stress–strain curves derived from spherical tip nanoindentation. Increased noise and oscillations were observed after the yield point, primarily due to greater uncertainty in contact area estimation during post-yield deformation. Note: red circles indicate the approximate indentation locations on the CT images and do not represent individual indentation points.

### 3.2. Elasto-plastic parameters

The elasto-plastic material properties calculated directly from the experiments and the equivalent parameters obtained through the inverse optimization procedure are presented in Fig 7. At each anatomical location, 3–5 replicate indentations were analyzed, and the reported material parameters are presented as the corresponding mean ± SD (i.e., Standard Deviation). These quantitative material parameters further demonstrate regional variation within the examined femur. The experimentally derived elastic moduli were generally higher than the optimized values, with a mean absolute symmetric difference of around 15.97%, indicating moderate agreement. This difference may result from estimating the modulus directly from the local slope near the apparent yield point. Near this transition, the slope is sensitive to smoothing, polynomial fitting, local oscillations, and strain-window selection, which may overestimate stiffness. For a representative cortical test, reducing the polynomial order from 12 to 8 changed the extracted elastic modulus by only 1.1%, while the apparent initial yield stress and strain varied by 9.1% and 8.3%, respectively. Additional details are provided in Section S1.1 of the S1 Appendix. Across the tested regions, the longitudinal cortical sample from the central diaphysis showed the highest elastic modulus, with an optimized value of 14.80 ± 0.49 GPa. Lower moduli in the radial and tangential directions reflected the orientation-dependent stiffness of cortical bone.

**Fig 7:**
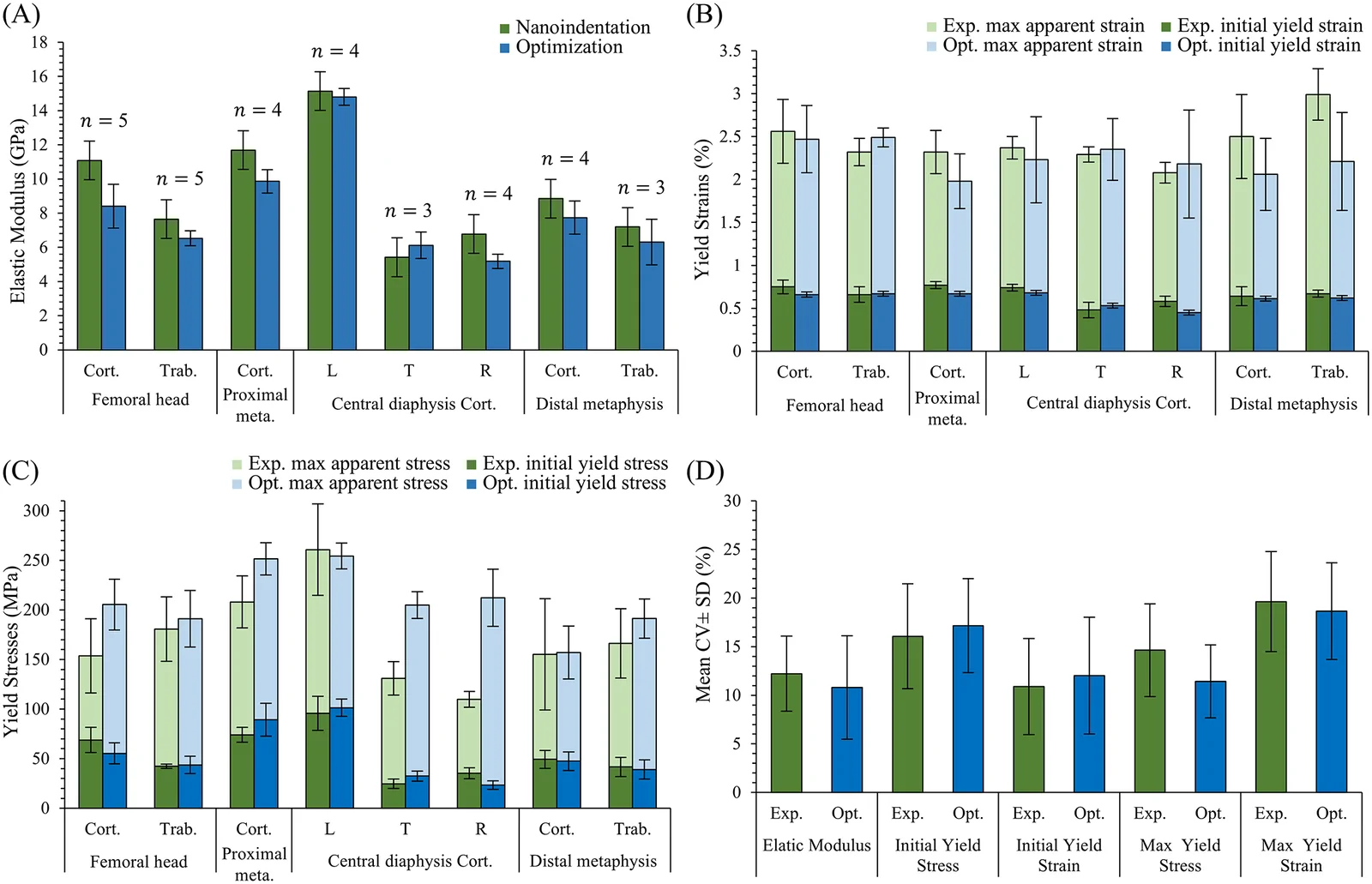
Elasto-plastic material properties obtained from the nanoindentation experiments and optimization framework. (A) elastic modulus, (B) initial and maximum apparent yield strains, (C) initial and maximum apparent yield stresses, and (D) average reproducibility of the experimental and optimized data expressed as the Coefficient of Variation, CV (%). The sample size for each location is indicated above the corresponding group in panel (A) and applies to panels (B) and (C). Abbreviations: Cort., cortical; Trab., trabecular; meta., metaphysis; L, longitudinal; T, tangential; R, radial; Exp., experimental; Opt., optimized; CV, coefficient of variation; SD, standard deviation.

The optimized initial apparent yield strains were clustered within a narrow range, mainly around 0.6–0.7%. This limited variation is expected because indentation strain represents a localized contact response rather than a true continuum strain field, with highly nonuniform strain concentrated beneath the spherical indenter. Thus, regional differences were more evident in modulus, yield stress, and post-yield response than in initial apparent yield strain. In contrast, the optimized maximum apparent yield strain showed greater variation, ranging from approximately 1.98% to 2.49%. The highest value was observed in the trabecular femoral head (2.49 ± 0.11%), consistent with the greater compliance and deformation capacity of trabecular bone. The optimized yield stress parameters showed clearer anatomical site dependence than the yield strains. The highest load-bearing response was observed in the longitudinal cortical direction of the central diaphysis, with an optimized initial apparent yield stress of 101.27 ± 8.71 MPa and a maximum apparent yield stress of 254.28 ± 13.05 MPa. The optimized maximum apparent stresses were generally higher than those estimated directly from the experiments, with a mean absolute symmetric difference of approximately 22.33%. This larger difference, compared with the elastic modulus estimates, reflects the greater dispersion and uncertainty in the post-yield region. The apparent experimental post-yield values should not be interpreted as direct counterparts of the optimized intermediate and terminal hardening parameters, which were inverse-identified from the force–displacement response.

To quantify the reproducibility and reliability of both the experimentally derived and optimized parameters, the Coefficient of Variation (CV) was calculated for each anatomical region using the corresponding mean and SD. The regional CV values were then summarized as the average CV ± SD across all regions, while the detailed region-specific CV values are provided in Table S2 and Table S3 of S1 Appendix. For the optimized parameters, the average CV values showed moderate variability, ranging from approximately 10.8% to 19.6%. The elastic modulus showed relatively consistent results, with average CV values of 10.81 ± 5.32% for the optimization framework. Yield-related parameters showed slightly higher variability, particularly the maximum apparent yield strain, with CV values of 18.65 ± 4.98% after optimization. The observed variability in the optimized values suggests that the framework preserves anatomical and tissue-level differences instead of excessively smoothing the material response.

The optimized plasticity parameters and MSE values are presented in Table 1. These parameters can be used in microscale computational frameworks to define the plastic region of the constitutive model and to support potential damage initiation criteria based on the maximum apparent stress and strain. For each anatomical region, the optimized elastic modulus and plastic stress–strain parameters were combined to define a region-specific elasto-plastic material model. The quality of the optimized parameter set was evaluated by comparing the experimental and optimized force–displacement responses. The region-specific MSE values ranged from 2.66 to 9.88 (mN)^2^, showing that the optimized models closely reproduced the experimental indentation responses. These errors were small compared with the corresponding force levels, indicating that the optimization procedure provided a reliable fit and could extract mechanically consistent elasto-plastic parameters for each region. Representative comparisons showed close agreement throughout the indentation depth and are provided in S2 Fig and Section S1.2 of the S1 Appendix.

**Table 1:** Optimized plasticity parameters from the GA-based inverse optimization framework.

Region	Bone Type	Optimized Plasticity Parameters						MSE (mN)^2^
		$\sigma_{y}^{0}$ (MPa)	$\epsilon_{y}^{0}\left(\%\right)$	$\sigma_{y}^{1}$ (MPa)	$\epsilon_{y}^{1}\left(\%\right)$	$\sigma_{y}^{2}$ (MPa)	$\epsilon_{y}^{2}\left(\%\right)$	
**FH**	Cortical	55.4 ± 10.6	0.66 ± 0.07	170.9 ± 21.4	1.44 ± 0.41	205.5 ± 25.6	2.47 ± 0.39	4.44 ± 2.83
	Trabecular	43.7 ± 8.7	0.67 ± 0.16	113.2 ± 27.4	1.26 ± 0.38	191.1 ± 28.6	2.49 ± 0.11	5.75 ± 3.31
**PM**	Cortical	66.4 ± 7.5	0.67 ± 0.06	162.6 ± 37.9	1.13 ± 0.08	235.4 ± 26.9	1.98 ± 0.32	2.75 ± 0.93
**CD**	Cortical – L	101.3 ± 8.7	0.68 ± 0.04	230.6 ± 27.5	1.17 ± 0.10	254.3 ± 13.0	2.23 ± 0.50	4.98 ± 0.56
	Cortical – T	32.5 ± 5.1	0.53 ± 0.05	108.8 ± 57.4	1.14 ± 0.15	204.9 ± 13.6	2.35 ± 0.36	2.66 ± 1.83
	Cortical – R	23.3 ± 4.2	0.45 ± 0.08	136.0 ± 29.4	1.39 ± 0.60	212.2 ± 28.9	2.18 ± 0.63	5.70 ± 4.03
**DM**	Cortical	47.5 ± 9.3	0.61 ± 0.09	138.5 ± 18.3	1.06 ± 0.22	157.1 ± 26.6	2.06 ± 0.42	5.28 ± 2.66
	Trabecular	39.1 ± 9.7	0.62 ± 0.03	144.0 ± 17.1	1.15 ± 0.16	191.3 ± 19.7	2.21 ± 0.57	9.88 ± 16.06

The parameters are reported for femoral head (FH), proximal metaphysis (PM), central diaphysis (CD), and distal metaphysis (DM), including initial yield, intermediate hardening, maximum apparent stress–strain values, and the corresponding fitting MSE. Note: For CD cortical samples, L = longitudinal, T = tangential, and R = radial.

For a representative cortical test, three independent GA runs with different initial populations produced comparable force–displacement fits (MSE = 1.67–1.91 (mN)^2^). The initial yield parameters and terminal hardening stress showed low variability (CV = 0.55–1.12%), whereas the intermediate hardening parameters showed greater variation (approximately 13%). More details are provided in Section S1.2 of the S1 Appendix.

### 3.3 Continuum-scale material properties

The *HU–ρ*_*app*_ relationship was estimated using two calibration phantoms scanned during μQCT with known densities of 0.25 and 0.75 g/cm^3^. The resulting calibration equation gives:


$$\begin{array}{c} \begin{array}{c} \rho_{app}=\text{7}\times {\text{1}\text{0}}^{-\text{4}}\;HU^{*}+\text{3}.\text{5}\text{7}\times {\text{1}\text{0}}^{-\text{2}}\; \end{array} \end{array}$$ (12)

where HU^*^ is the protocol-dependent *HU*. Although the $HU^{*}-\rho_{app}$ calibration is protocol-dependent, converting HU^*^ to *ρ*_*app*_ allows the homogenized effective modulus, *E*_*eff*_, to be expressed through an $E-\rho_{app}$ relationship, reducing *direct dependence* on raw image intensity. The trabecular RVEs were initially assigned *E* = 6.53 GPa and *ν* = 0.3, based on the optimized properties of the femoral head trabecular samples. For RVE_41_ and RVE_42_, extracted from the distal metaphysis, *E* = 7.74 GPa and *ν* = 0.3 were used to represent the relatively porous cortical–trabecular transition region. The maximum directional effective modulus obtained from homogenization was selected as the primary load-bearing modulus and correlated with the corresponding apparent density values to define the $E_{eff}^{trab}-\rho_{app}$ relationship for material assignment. For both trabecular and cortical bone, apparent density was calculated from 2 × 2 × 2 mm^3^ μQCT-derived sub-volumes. These correlations are illustrated in Fig 8. Detailed homogenization results are provided in S1 Appendix (Table S4).

**Fig 8:**
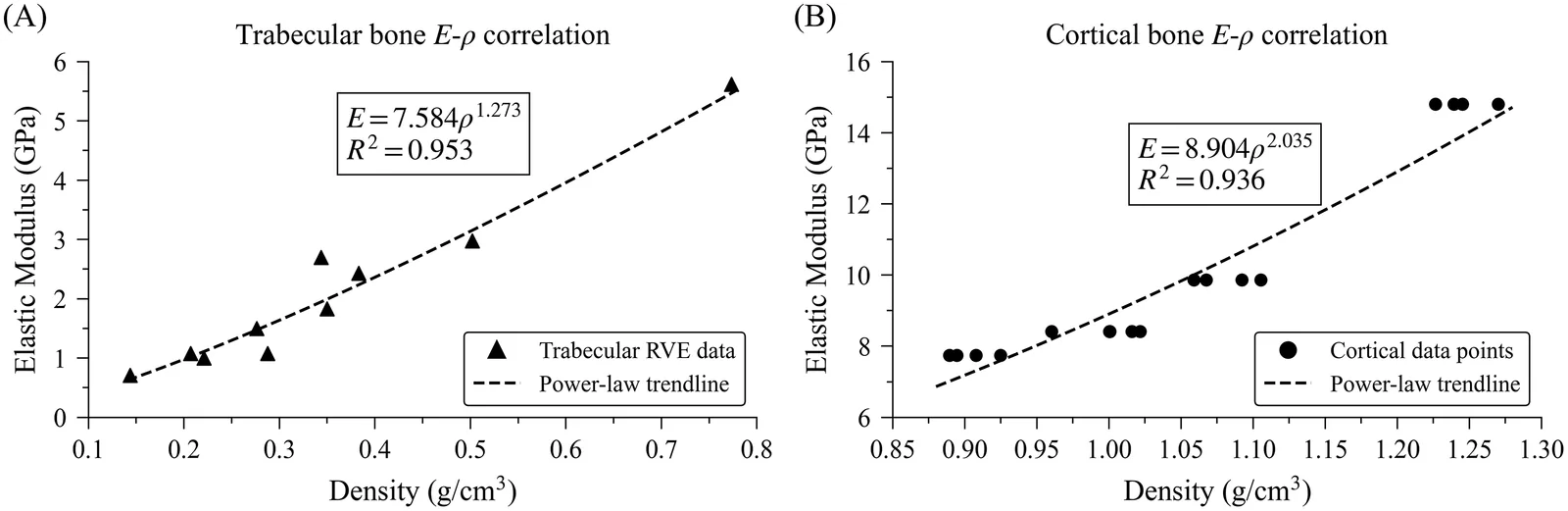
Modulus–density correlations derived through power-law curve fitting. (A) Trabecular and (B) cortical bone regions. Trabecular data points represent μQCT-derived RVE apparent densities paired with effective moduli obtained from homogenization. Cortical data points represent μQCT-derived apparent densities paired with the corresponding region-specific average elastic moduli obtained from the optimization. The resulting fitted relationships were used to define compartment-specific continuum-scale material properties.

The fitted $E-\rho_{app}$ relationships were then used to assign spatially varying elastic properties to the trabecular- and cortical-dominant compartments. Separate correlations were defined for each compartment to account for differences in their effective $E-\rho_{app}\;$response. The final compartment-specific elastic modulus map was defined as:


$$\begin{array}{c} \begin{array}{c} E\left(\rho_{app}\right)=\left\{ \begin{array}{cc} \text{7}.\text{5}\text{8}\text{4}\;\rho_{app}^{\text{1}.\text{2}\text{7}\text{3}} & \text{ trabecular}\\ \text{8}.\text{9}\text{0}\text{4}\;\rho_{app}^{\text{2}.\text{0}\text{3}\text{5}} & \text{cortical} \end{array}\right.\; \end{array} \end{array}$$ (13)

For this study, considering the CT imaging protocol and the observed HU^*^ ranges, Eqs 12 and 13 were combined to define the elastic modulus map as a function of HU^*^. Voxels with HU^*^ ≥ 500 were considered cortical-dominant regions, while voxels with HU^*^ < 500 were considered trabecular-dominant regions. Therefore, the elastic modulus map was defined as:


$$\begin{array}{c} \begin{array}{c} E\left(HU^{*}\right)=\left\{ \begin{array}{cc} \text{7}.\text{5}\text{8}\text{4}{\left(\text{7}\times {\text{1}\text{0}}^{-\text{4}}HU^{*}+\text{3}.\text{5}\text{7}\times {\text{1}\text{0}}^{-\text{2}}\right)}^{\text{1}.\text{2}\text{7}\text{3}} & HU^{*}<\text{5}\text{0}\text{0}\\ \text{8}.\text{9}\text{0}\text{4}{\left(\text{7}\times {\text{1}\text{0}}^{-\text{4}}HU^{*}+\text{3}.\text{5}\text{7}\times {\text{1}\text{0}}^{-\text{2}}\right)}^{\text{2}.\text{0}\text{3}\text{5}} & HU^{*}\geq \text{5}\text{0}\text{0} \end{array}\right.\; \end{array} \end{array}$$ (14)

In addition to elastic mapping, the optimized plasticity parameters were interpolated as functions of E(HU^*^) to assign spatially consistent elasto-plastic properties across the finite element model. Accordingly, the yield and post-yield parameters were assigned based on the local elastic modulus obtained from the compartment-specific E(HU^*^) relationship, allowing both elastic and plastic responses to reflect regional tissue heterogeneity. The resulting material definitions therefore included *E* together with *σ*_*y*_^*0*^, *ε*_*y*_^*0*^, *σ*_*y*_^*1*^, *ε*_*y*_^*1*^, *σ*_*y*_^*2*^, and *ε*_*y*_^*2*^. The complete elasto-plastic parameter sets assigned to the HU^*^ intervals are summarized in Table 2. For each HU^*^ interval, a representative elastic modulus was calculated from Eq 14 using the midpoint HU^*^ value of that interval, and the corresponding plasticity parameters were obtained through the *E*-dependent interpolation described in Eq 11.

**Table 2:** Elasto-plastic material properties for continuum FEA using the compartment-specific E(HU^*^) relationship and E-dependent interpolation of the optimized plasticity parameters.

HU^*^ range	Bone type	E (GPa)	$\sigma_{y}^{0}\;$(MPa)	$\varepsilon_{y}^{0}$(%)	$\sigma_{y}^{1}$ (MPa)	$\varepsilon_{y}^{1}$(%)	$\sigma_{y}^{2}$ (MPa)	$\varepsilon_{y}^{2}$(%)
80–289	Trabecular HU^*^ < 500	0.76	11.8	1.073	18.8	1.382	24.0	2.057
290–499		1.72	18.5	1.075	30.4	1.386	38.8	2.062
500–680	Cortical HU^*^ ≥ 500	1.74	18.8	1.076	30.9	1.386	39.4	2.072
681–861		2.89	31.3	1.080	52.4	1.392	66.9	2.073
862–1041		4.33	46.8	1.081	74.7	1.393	95.1	2.073
1042–1222		6.06	65.6	1.082	103.4	1.393	131.8	2.080
1223–1402		8.10	87.9	1.085	138.1	1.398	176.5	2.080
1403–1583		10.43	113.1	1.084	177.9	1.398	227.1	2.083
1584–1763		13.06	142.2	1.088	207.1	1.403	239.4	2.087
1764–1950		16.00	175.1	1.095	237.6	1.411	249.2	2.100

To demonstrate continuum-scale material assignment, the canine femur RVEs were color-mapped using the indentation-derived bone tissue modulus values as inputs for homogenization, as shown in Fig 9A. The corresponding continuum-scale elements were then assigned the homogenized properties obtained for each RVE. The effective elastic modulus of the homogenized continuum elements was slightly lower than the tissue-level modulus because it accounts for the influence of porosity and trabecular architecture. The whole femur was then segmented from µQCT images, and an adaptive mesh with an element size of 1.0 mm was generated to depict material assignment, as shown in Fig 9B. For each element, the average grayscale value of the µQCT voxels contained within that element was approximated as the equivalent CT grayscale value for material assignment. Eq 14 was then used to assign compartment-specific elastic modulus to the continuum-scale canine femur model, while the corresponding plasticity parameters were assigned using the *E*-dependent interpolation procedure described in Eq 11 and Table 2.

**Fig 9:**
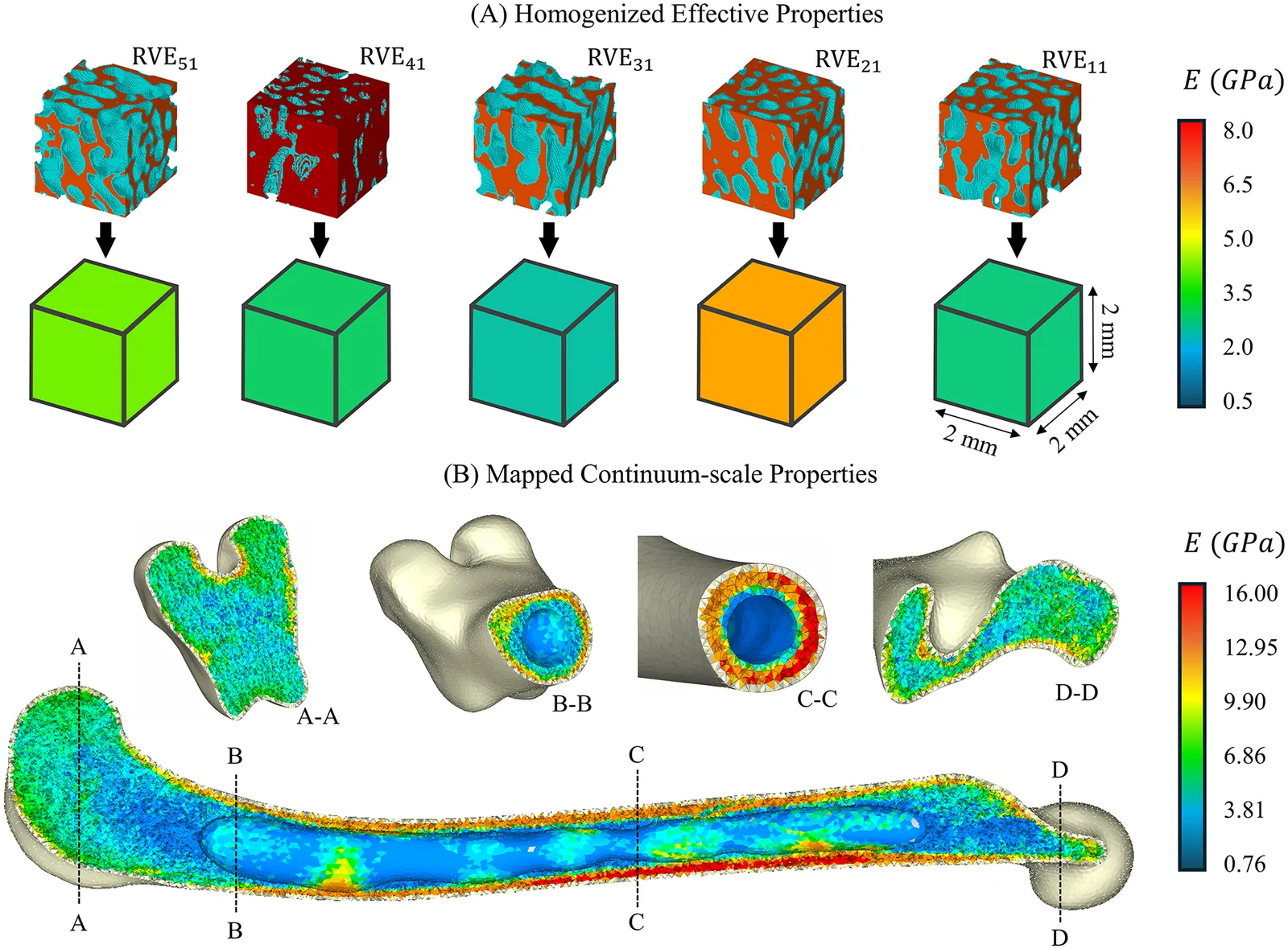
RVE homogenization and continuum-scale material assignment in the canine femur. (A) RVEs extracted from different anatomical regions of the femur, color-mapped using the indentation-derived tissue-level elastic modulus and homogenized to obtain the corresponding effective elastic moduli, $E_{eff}^{trab}$. (B) Continuum-scale femur mesh used to illustrate compartment-specific material assignment based on the $HU^{*}-\rho_{app}$ relationships. Trabecular pores were filled for continuum modeling, and a 1-mm cortical shell is shown in beige (E ≈ 10.60 GPa). Teal-blue regions in the RVEs are visualization artifacts at pore–bone transitions only; each RVE was assigned a single modulus before homogenization.

Although the spatial map in Fig 9 is visualized using elastic modulus, each HU^*^ interval represents the corresponding complete elasto-plastic material parameter set reported in Table 2.

To demonstrate the broad applicability of the approach, the proposed material-mapping was applied to other clinical CT-based models of the canine humerus, tibia, and radius for macroscale FEA simulations, as shown in Fig 10. Since the clinical CT voxel size in these datasets was approximately 500–900 µm, the trabecular microarchitecture could not be explicitly resolved. Accordingly, trabecular regions were represented using equivalent continuum properties rather than direct modeling of individual trabeculae. For each HU^*^ interval, the elastic modulus was determined from the compartment-specific relationship, and the corresponding yield and post-yield parameters were assigned using the *E*-dependent interpolation procedure described in Eq 11 and Table 2.

**Fig 10:**
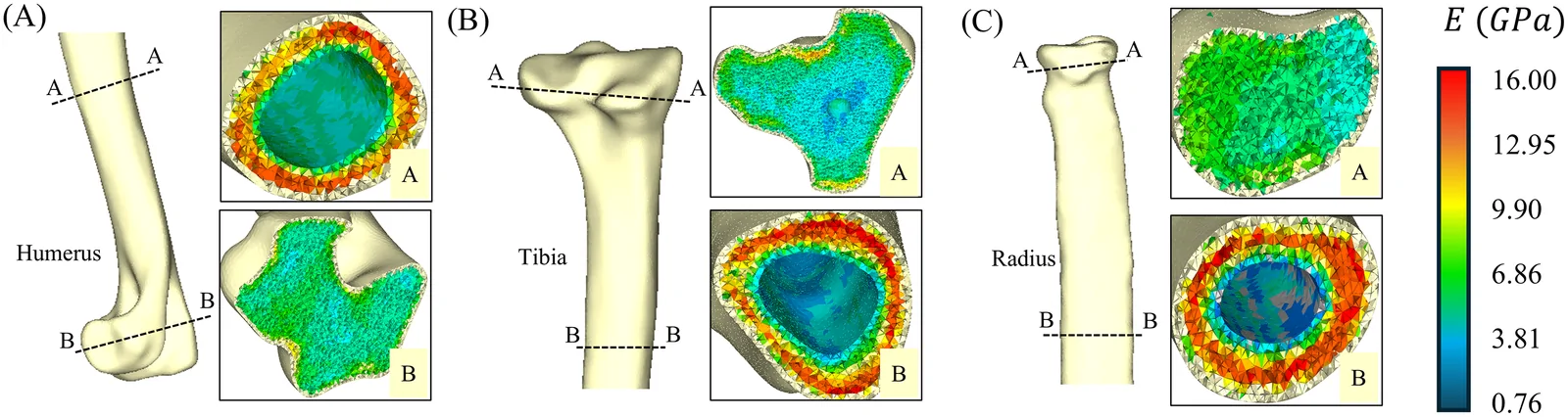
Application of the proposed compartment-specific material-mapping approach to clinical CT-based models of the canine (A) humerus, (B) tibia, and (C) radius. Representative sections illustrate spatial variation in assigned elastic modulus, with higher-modulus cortical regions and trabecular regions represented using a continuum mesh rather than computationally expensive fine-scale trabecular modeling.

The resulting whole-bone meshes contained 782,856 tetrahedral elements for the humerus (0.8 mm element size), 671,302 for the tibia (0.8 mm), and 985,394 for the radius (0.85 mm). The representative femur model contained 1,121,156 elements (1.0 mm).

## 4. Discussion

The integrated experimental–numerical pathway developed in this study proposes a material mapping approach for assigning site- and compartment-specific elasto-plastic properties to bone in conventional patient-specific FEA. The results show that spherical nanoindentation can capture tissue-level mechanical responses across different anatomical sites, while the GA-based inverse optimization procedure reduces reliance on post-yield contact-area estimation. This provides more stable and mechanically consistent material parameters, particularly in the post-yield region, compared with direct interpretation of indentation-derived stress–strain curves. By combining the optimized tissue-scale properties with RVE homogenization and *E–ρ*_*app*_ correlations, the proposed pathway links microscale measurements to continuum-scale material maps for patient-specific FEA models reconstructed from clinical CT images, where trabecular microarchitecture cannot be explicitly resolved.

The observed spatial variation emphasizes the importance of comprehensive region-specific characterization, as different anatomical sites are subjected to distinct loading modes, loading directions, and functional demands. These local mechanical environments can influence tissue organization, density, mineralization, and overall mechanical response [53–55]. Therefore, testing multiple regions provides a more representative basis for capturing bone heterogeneity and developing spatially informed material properties for FEA. Another major observation was the increased dispersion and oscillatory behavior of the calculated stress–strain curves after the onset of yielding. While the initial elastic region showed relatively smoother and more consistent behavior, the yield and post-yield regions exhibited greater fluctuations. These oscillations may arise from a combination of localized plastic deformation, microstructural heterogeneity, and uncertainties in contact-area estimation. As discussed by Pathak and Kalidindi [34], pile-up and sink-in effects, changes in contact radius, non-ideal indenter geometry, and specimen surface conditions can influence the calculated indentation strain and stress after plastic deformation begins. These effects introduce uncertainty in the extraction of plasticity parameters and further support the use of a numerical framework to regularize the post-yield response.

Epoxy embedding may introduce local mechanical constraint or support to the bone tissue, particularly in porous trabecular regions where resin infiltrates adjacent pores. However, the magnitude of this effect depends on both the embedding condition and the indentation sampling volume. Bushby et al. [53] showed that hydration and PMMA embedding can alter the measured indentation modulus of bone, while also demonstrating that larger-radius spherical indenters and higher loads increase the sampled material volume and reduce sensitivity to localized near-surface effects. In the present study, a 158 μm diameter spherical indenter was used, and indentations were positioned within the bone tissue and away from visible bone–epoxy interfaces, which was expected to reduce direct surface and boundary effects. Mittra et al. [20] further reported that substantial variations in epoxy stiffness did not significantly affect the measured elastic modulus of canine trabecular bone, although hardness was influenced. Nevertheless, local confinement or mechanical support resulting from epoxy infiltration, particularly around trabecular struts, cannot be completely excluded as a source of measurement uncertainty.

Previous canine bone studies have mainly focused on cortical bone and have commonly reported elastic modulus, while trabecular bone properties and post-yield behavior remain less characterized. The longitudinal cortical diaphyseal modulus obtained in the present study (14.80 ± 0.49 GPa) was comparable to values previously reported for hydrated canine femoral cortical bone by Lee et al. [18] (13.41 ± 5.70 GPa) and Autefage et al. [54] (15.60 ± 2.6 GPa), and also fell within the range reported by Luo et al. [19] (approximately 15–22 GPa). Although the elastic modulus derived in the current study is generally comparable to reported hydrated values, this agreement may still be influenced by specimen-specific factors, including bone mass density and anatomical region. Hydration condition is nevertheless known to influence bone stiffness; for example, Lee et al. [55] reported elastic moduli of approximately 22–25 GPa for dry canine femoral cortical bone compared with approximately 13–15 GPa under wet conditions. The relatively lower modulus obtained in the present study under dry conditions may reflect differences in indentation geometry, specimen preparation, and stress–strain analysis. Therefore, comparisons with literature values should be interpreted in the context of these methodological differences. The above-mentioned studies therefore establish a useful reference range for the elastic modulus of hydrated canine femoral cortical bone; however, their scope was primarily limited to elastic characterization rather than full elasto-plastic stress–strain determination. Furthermore, Luo et al. [19] identified trabecular bone measurement as a major limitation, emphasizing the difficulty of fully characterizing both cortical and trabecular compartments. The variation in modulus values reported by Lin et al. [56] for canine femoral cortical bone (25.7 GPa) and by Mittra et al. [20] for dry canine femoral trabecular bone (12.97 ± 4.15 GPa) may be partly explained by differences in anatomical site, specimen condition, indenter geometry, and analysis method, particularly when modulus is extracted from the unloading response. Although lower than the value reported by Mittra et al. [20], the trabecular modulus obtained from the present dry specimens was of a similar magnitude. Overall, these comparisons suggest that the cortical elastic moduli obtained in this study are consistent with reported hydrated canine bone values, while the present work extends previous studies by incorporating trabecular regions and deriving elasto-plastic parameters.

As discussed previously, the benefit of the inverse-identification framework is particularly important for the post-yield response. The indentation-derived stress–strain curve provides initial estimates of the elastic modulus and apparent yield point; however, its direct interpretation becomes increasingly uncertain after yielding because plastic deformation alters the evolving contact geometry. The GA-based inverse optimization procedure therefore uses the experimental load–depth response to refine the constitutive parameters and identify the intermediate and terminal hardening points within the calibrated indentation range. This provides a complete elasto-plastic material description without prescribing an arbitrary post-yield hardening response. The repeatability assessment further showed that the initial yield parameters and terminal hardening stress were consistently identified across independent GA runs, whereas the intermediate hardening point was less tightly constrained. This indicates good robustness of the principal constitutive parameters, although some non-uniqueness remains in the intermediate hardening description.

Another key advantage of this framework is that it reduces the need to explicitly model trabecular microarchitecture in macroscale bone FEA. By deriving density-based elasto-plastic material maps, the effective continuum response of trabecular bone can be incorporated without requiring complex reconstruction from µQCT data or computationally expensive trabecular meshing. This is particularly important for patient-specific FEA derived from clinical images, where rapid model generation, computational efficiency, and numerical stability are essential. The resulting material maps can support higher-fidelity estimation of damage initiation, fracture risk, and load-bearing capacity. The power-law model, $E=\alpha \;\rho_{app}^{\beta }$, used to fit the developed $E-\rho_{app}$ relationship is consistent with formulations reported in previous canine bone studies [25,57]. However, because reported modulus–density relationships vary with anatomical site, density definition, and testing method, the present relationships should be interpreted within the anatomical and experimental conditions investigated in this study [25,58]. In future studies, these relationships may also be adapted to degraded bone conditions, such as osteosarcoma-associated bone loss or osteoporosis, provided that appropriate density- and pathology-specific correction factors are incorporated.

Figs 9 and 10 demonstrated the application of the proposed material-mapping framework to whole-bone continuum FEA models in which trabecular microarchitecture is represented through homogenized material properties rather than explicit trabecular geometry. The resulting models contained approximately one million tetrahedral elements with element sizes of 0.8–1.0 mm. High-resolution FEA models that explicitly resolve individual trabecular architecture require substantially finer discretization and several million elements. For instance, Van Rietbergen et al. [59] reported approximately 7.6 million elements for a canine proximal femur, while approximately 2–21 million elements have been reported for localized human and murine bone regions [60,61]. This comparison illustrates the computational advantage of representing trabecular bone through continuum-scale homogenized properties rather than explicitly resolving individual trabeculae in whole-bone FEA studies.

Although this study was motivated by the limited availability of elasto-plastic material data for canine long bones, the proposed framework is not limited to a single species, anatomical site, or bone type. The same methodological workflow can be adapted to other bones, species, age groups, or pathological conditions, provided that suitable imaging and experimental data are available. In this context, the canine femur is presented as a representative application of the framework rather than the limit of its broader use. The application to the humerus, tibia, and radius demonstrates how compartment-specific continuum material maps can be implemented in whole-bone models without explicitly resolving trabecular microarchitecture, thereby maintaining a computational scale suitable for nonlinear whole-bone FEA.

As a proof-of-concept study, the present framework was developed using a selected set of anatomical regions and a single canine femur. Trabecular characterization was focused on the femoral head and distal metaphysis, where sufficiently prominent trabecular architecture was available for nanoindentation. In addition, the RVEs were selected to represent regional trabecular architecture rather than being spatially co-localized with individual indentation sites; therefore, a direct one-to-one correspondence between local tissue properties and specific RVE architecture was not established. The homogenization procedure captured the architecture-dependent elastic response through the effective stiffness tensor, while the progressive trabecular yielding and load redistribution were incorporated at the continuum level through the subsequent *E*-dependent interpolation of yield and post-yield parameters rather than being explicitly homogenized. Because the study was based on a single femur, the observed regional differences should be interpreted as within-specimen variations. Future studies incorporating additional trabecular regions, larger and well-characterized cohorts, and more comprehensive directional testing will be needed to evaluate the generalizability of the proposed material relationships and further develop the framework toward a fully orthotropic elasto-plastic characterization.

## 5. Conclusion

Clinical and preclinical assessments increasingly rely on numerical simulations such as FEA to estimate bone load-bearing capacity under different conditions, including pathological cases and the evaluation of biomedical devices intended to reinforce bone. Although FEA is a well-established tool in biomechanics, its clinical and translational use requires material modeling strategies that are both reliable and computationally efficient. The framework proposed in this study addresses this need by translating tissue-scale mechanical properties into continuum-scale material inputs that can be directly utilized in whole-bone macroscale FEA models. This is particularly important for canine long bones, where the literature still lacks sufficient experimentally derived material data for veterinary FEA applications, although the same concept may also be extended to other species, including humans.

Many CT-based bone models assign material properties using global values or empirical relationships with grayscale intensity and density. However, these approaches may overlook regional variations and the mechanical influence of trabecular microarchitecture. The proposed framework addresses this gap by incorporating homogenized trabecular properties into continuum-scale material maps, allowing whole-bone FEA models to account for trabecular architecture without explicitly modeling each trabecula. This strategy provides a more practical and mechanically representative material input for heterogeneous bone simulations. Although spherical nanoindentation introduces several challenges, particularly the noisy interpretation of post-yield stress–strain behavior, the proposed GA-based inverse optimization framework provided a practical solution for extracting more stable and mechanically consistent elasto-plastic properties. This is especially important because post-yield behavior plays a key role in predicting damage initiation and progression in bone mechanics. The application of these correlations to four different canine long bones further demonstrates their potential as practical constitutive relationships linking density to material properties in whole-bone FEA. Because these relationships were developed from a single canine femur, further validation across additional specimens is necessary to assess their generalizability, although the same procedure can be applied. Moreover, a direct comparison between the optimized material maps and conventional CT-based material assignments would provide an important next step for evaluating their influence on whole-bone mechanical predictions. Overall, this framework extends the use of experimental data beyond simple property reporting and provides heterogeneous elasto-plastic material maps for future patient-informed biomechanical simulations.

## Supporting information

S1 Appendix Supplementary methods and results. Additional details regarding spherical nanoindentation calibration and stress–strain formulation, GA-based inverse optimization, RVE reconstruction from μQCT data and homogenization, variability of experimental and optimized material parameters, and homogenized orthotropic properties of trabecular RVEs.
(DOCX)

S1 FigIllustration of the experimental and numerical definition of the elasto-plastic parameters.(A) representative nanoindentation force–displacement response; (B) corresponding indentation stress–strain curve showing the experimentally estimated initial apparent yield point; and (C) schematic representation of the three stress–strain control points used in the inverse optimization, where $\left(\sigma_{y}^{\text{0}},\varepsilon_{y}^{\text{0}}\right)$ represents the initial apparent yield point, $\left(\sigma_{y}^{\text{1}},\varepsilon_{y}^{\text{1}}\right)$ is the intermediate hardening control point, and $\left(\sigma_{y}^{\text{2}},\varepsilon_{y}^{\text{2}}\right)$ is the terminal hardening point within the calibrated strain range.(TIF)

S2 FigComparison of experimental and optimized FEA responses for representative cortical samples.(A–C) Experimental and optimized FEA force–displacement curves showing agreement throughout the indentation depth. (D–F) Corresponding apparent indentation stress–strain responses with the inverse-identified constitutive control points $\left(\sigma_{y}^{\text{0}},\;\varepsilon_{y}^{\text{0}}\right)$, $\left(\sigma_{y}^{\text{1}},\;\varepsilon_{y}^{\text{1}}\right)$, and $\left(\sigma_{y}^{\text{2}},\;\varepsilon_{y}^{\text{2}}\right)$. The second and third control points are inverse-identified post-yield parameters and are not expected to coincide directly with the apparent experimental stress–strain response because post-yield contact-area estimation becomes increasingly uncertain.(TIF)
