# Supplementary material for: An integrated experimental–computational framework for elasto-plastic characterization and spatial mapping of canine bone properties
Source: PLoS One. 2026 Oct 23;21(10):e0361283. doi: 10.1371/journal.pone.0361283 (PMC13693985; doi:10.1371/journal.pone.0361283)
Supplement: Supplementary file 1 [file pone.0361283.s001.docx]

S1 Appendix

S1.1 Spherical nanoindentation calibration and stress–strain formulation

The spherical tip should be calibrated before conducting nanoindentation experiments. This step is crucial to ensure the accuracy and reliability of the results. Through calibration, the relationship between the contact depth, $h_{c}$, and the contact radius, $a$, is established as:

$$\begin{aligned} a = \sqrt{\frac{1}{\pi}\sum_{0}^{n} C_{n}{h_{c}}^{2/2^{n}}} \#\left( S1.1 \right) \end{aligned}$$

The area function coefficients, $C_{n}$, are determined during the calibration process using the Nano Indenter XP software by performing tests on fused silica with a nominal modulus of 72 GPa and Poisson’s ratio of 0.18. For the spherical indenter, the KLA software provided three area-function coefficients (*C_0_*, *C_1_*, and *C_2_*) from the fused-silica calibration. These coefficients were typically sufficient to describe the relationship between contact area and contact depth over the calibrated indentation range and to account for deviations of the actual tip geometry from an ideal spherical shape. Additional higher-order terms were therefore not required and could unnecessarily increase model complexity, potentially leading to overfitting or numerical instability. The calculated area function coefficients for the sapphire tip in this study are shown in Table S1.

Table S1. Area coefficients derived from calibration with fused silica. The coefficients *C_0_*, *C_1_*, and *C_2_* define the calibrated projected contact-area function used for the spherical indenter.

| **Coefficient** | $C_{0}$ | $C_{1}$ | $C_{2}$ |
| --- | --- | --- | --- |
| Value | $-\pi$ | 279128.37 | 1676243.14 |

As stated in Section 2.2, the reduced modulus, *E^*^*, is calculated as:

$$\begin{aligned} E^{*}=\left[ \frac{1-\nu^{2}}{E}+\frac{1-\nu_{i}^{2}}{E_{i}} \right]^{-1}\#\left( S1.2 \right) \end{aligned}$$

where *E* and *ν* are the elastic modulus and Poisson’s ratio of the sample, respectively, while *E_i_* = 345 GPa and *ν_i_* = 0.29 are the corresponding properties of the indenter. $E^{*}$ can be found using the total indentation depth, *h_t_*, and contact depth, *h_c_*, through:

$$\begin{aligned} h_{t}-h_{c}=\frac{1}{2}\left( \frac{3P}{4E^{*}} \right)^{\frac{2}{3}}\left( \frac{1}{R} \right)^{\frac{1}{3}} \#\left( S1.3 \right) \end{aligned}$$

where *P* is the measured indentation load and *R* is the spherical indenter radius.

This relationship establishes a connection between the contact depth and the elastic displacement of the sphere into the material. The indenter radius is related to the contact depth and projected contact radius. By combining Eqs S1.2 and S1.3, the elastic modulus can be determined as follows,

$$\begin{aligned} E=\left( 1-\nu^{2} \right)\cdot\left[ \frac{4}{3P}\left( \frac{2\left( h_{t}-h_{c} \right)}{\left( \frac{2h_{c}}{a^{2}+h_{c}^{2}} \right)^{1/3}} \right)^{3/2}-\frac{1-\nu_{i}^{2}}{E_{i}} \right]^{-1} \#\left( S1.4 \right) \end{aligned}$$

The representative indentation stress was calculated from the corrected indentation load and projected contact area as:

$$\begin{aligned} \sigma=\frac{\psi P}{\pi a^{2}}=\frac{0.9\left( \frac{h_{t}}{h_{c}}-1 \right)P}{\pi a^{2}} \#\left( S1.5 \right) \end{aligned}$$

where $\psi= 0.9 (ht/hc-1)$ is a depth-dependent correction factor; therefore, *ψP* represents the corrected indentation load. Once the indentation stress, $\sigma$, and elastic modulus of the material, $E$, are known, the indentation strain can be obtained using Hooke’s law assuming linear elasticity. In other words, the strain can be obtained using ${\varepsilon=E}^{-1}\sigma$. Hence, the expression of indentation strain is given as:

$$\begin{aligned} \varepsilon=\left( \frac{0.9P}{\pi a^{2}\left( 1-\nu^{2} \right)}\left( \frac{h_{t}}{h_{c}}-1 \right) \right)\left( \frac{4}{2.7P\left( \frac{h_{t}}{h_{c}}-1 \right)}\left( \frac{2\left( h_{t}-h_{c} \right)}{\left( \frac{2h_{c}}{a^{2}+h_{c}^{2}} \right)^{1/3}} \right)^{3/2}-\frac{1-\nu_{i}^{2}}{E_{i}} \right) \#\left( S1.6 \right) \end{aligned}$$

Using these expressions, the stress and strain generated during the nanoindentation process can be calculated.

To assess the sensitivity of the extracted elastic modulus and apparent initial yield point to the polynomial fit, the analysis was repeated for a representative central-diaphysis cortical test using polynomial orders of 12, 10, and 8 (Table S2). The elastic modulus varied by only 1.1%, while the apparent yield stress and strain varied by 9.1% and 8.3%, respectively. However, $\sigma_{y,ind}^{0}/\varepsilon_{y,ind}^{0}$varied by less than 0.8%, indicating that the estimated elastic response remained essentially unchanged. Lower-order fits shifted the detected yield point slightly because of increased smoothing near the elastic–plastic transition.

Table S2. Sensitivity of the extracted elastic modulus and apparent initial yield parameters to the polynomial fitting order for a representative cortical test.

| **N (order of polynomial)** | **E (GPa)** | $\boldsymbol{\sigma}_{\boldsymbol{y,ind}}^{\boldsymbol{0}}$ **(MPa)** | $\boldsymbol{\varepsilon}_{\boldsymbol{y,ind}}^{\boldsymbol{0}}\boldsymbol{(\%)}$ |
| --- | --- | --- | --- |
| 12 | 7.57 | 61.95 | 0.96 |
| 10 | 7.51 | 58.50 | 0.91 |
| 8 | 7.49 | 56.33 | 0.88 |

S1.2 GA-based inverse optimization procedure

GA is a global optimization technique based on the principles of natural selection and evolution. This technique operates on a population of potential solutions, progressively improving them through a process akin to biological evolution. The typical steps in GA include initializing a population, evaluating their fitness, selecting the fittest individuals, and applying crossover and mutation operators to create new populations. This iterative process continues until a predefined termination criterion is met. The design variables for the optimization problem are $\sigma_{y}^{0},\varepsilon_{y}^{0},\sigma_{y}^{1},\varepsilon_{y}^{1},\sigma_{y}^{2},\varepsilon_{y}^{2}$. S1 Fig illustrates the relationship between the experimental nanoindentation response and the three stress–strain control points used in the inverse optimization. In the ABAQUS implementation, the elastic modulus is calculated by $E ={\sigma_{y}^{0}}/{\varepsilon_{y}^{0}}$ and the first plastic strain value is set to 0. Therefore, when defining material properties, the plastic strains are entered as 0, $\varepsilon_{y}^{1}-\varepsilon_{y}^{0}$ and $\varepsilon_{y}^{2}-\varepsilon_{y}^{0}$, respectively. The locations and roles of the three stress–strain control points used to define the isotropic hardening response are illustrated in S1 Fig.

The lower and upper bounds of the optimization were defined using the initial yield stress and strain estimated from the indentation stress–strain response. The initial yield stress $\sigma_{y,ind}^{0}$ and strain $\varepsilon_{y,ind}^{0}$ were used to bound $\sigma_{y}^{0}$ and $\varepsilon_{y}^{0}$ within 90–110% of their estimated values:

$$\begin{aligned} \begin{matrix} 0.9 \sigma_{y,ind}^{0}\leq\sigma_{y}^{0}\leq1.1\sigma_{y,ind}^{0} \\ {0.9 \varepsilon}_{y,ind}^{0}\leq\varepsilon_{y}^{0}\leq1.1\varepsilon_{y,ind}^{0} \end{matrix} \#\left( S1.7 \right) \end{aligned}$$

For the subsequent post-yield points shown in S1 Fig, the lower limits were set to the initial yield stress and strain, while the upper limits were set to 300 MPa for stress and 3% for strain. It should be noted that ABAQUS linearly extrapolates the hardening curve beyond the last defined point without imposing a saturation stress. However, due to the limit, the terminal strain ε_y_^2^ in all simulations must remain bounded at or below 3% strain. Therefore, the extrapolated portion was not invoked and does not affect the fitted stress–strain response. The GA was initialized with a population size of 50. To improve the distribution of the initial candidates across the search space, 90% of the initial population was generated using Latin Hypercube Sampling between the defined lower and upper bounds. The remaining 10% was generated using known initial yield estimates with perturbations of 0, $\pm1\%$, $\pm2.5\%$, $\pm5\%$, and $\pm10\%$ around $\sigma_{y,ind}^{0}$ and $\varepsilon_{y,ind}^{0}$. Any candidate violating the monotonicity constraints was rejected and resampled.

The loading phase of the FEA nanoindentation test was simulated using the following steps:

- The maximum penetration depth ($h_{exp}^{max}$) from the experimental data is applied to the reference point.
- The simulation is performed under static conditions, incorporating nonlinear geometric effects.
- The ABAQUS solver incrementally increases the applied displacement from 0 to $h_{exp}^{max}$.
- During simulation, reaction forces at the reference point are written in the output database file (.odb).

After running the simulation, the ABAQUS .odb file is read and reaction force ($\boldsymbol{P}_{opt}$) at the RP is extracted for all displacement ($\boldsymbol{h}_{opt})$ increments. The $\boldsymbol{P}_{opt}-\boldsymbol{h}_{opt}$ relationship is then fitted with a 12^th^-degree polynomial. Similarly, the loading part of the force ($\boldsymbol{P}_{exp}$) and displacement ($\boldsymbol{h}_{exp}$) from the experimental data are recorded and fitted with a 12^th^-degree polynomial. Higher-order polynomials are used in both cases to ensure an accurate fit.

To evaluate the consistency of the identified material parameters, a representative central-diaphysis cortical specimen tested in the tangential direction was selected for repeated optimization. The GA procedure was run three additional times using independently generated initial populations, and the resulting parameter sets were compared with the originally reported solution, as summarized in Table S3.

Table S3. Repeatability of GA-based inverse identification for a representative cortical test using different initial populations.

| **Repeat** | $\boldsymbol{\sigma}_{\boldsymbol{y}}^{\boldsymbol{0}}$ **(MPa)** | $\boldsymbol{\varepsilon}_{\boldsymbol{y}}^{\boldsymbol{0}}\boldsymbol{(\%)}$ | $\boldsymbol{\sigma}_{\boldsymbol{y}}^{\boldsymbol{1}}$ **(MPa)** | $\boldsymbol{\varepsilon}_{\boldsymbol{y}}^{\boldsymbol{1}}\boldsymbol{(\%)}$ | $\boldsymbol{\sigma}_{\boldsymbol{y}}^{\boldsymbol{2}}$ **(MPa)** | $\boldsymbol{\varepsilon}_{\boldsymbol{y}}^{\boldsymbol{2}}\boldsymbol{(\%)}$ | **MSE (mN)^2^** |
| --- | --- | --- | --- | --- | --- | --- | --- |
| 1 | 32.84 | 0.478 | 144.30 | 0.911 | 189.92 | 2.667 | 1.67 |
| 2 | 33.18 | 0.483 | 158.88 | 1.180 | 194.77 | 2.325 | 1.91 |
| 3 | 33.46 | 0.479 | 126.00 | 0.930 | 191.79 | 2.380 | 1.69 |
| Reported | 32.67 | 0.482 | 172.20 | 1.100 | 190.55 | 1.939 | 1.72 |

The initial yield parameters were highly consistent across the four solutions, with Coefficients of Variation (CV) of 1.07% for $\sigma_{y}^{0}$ and 0.55% for $\varepsilon_{y}^{0}$. The terminal hardening stress $\sigma_{y}^{2}$ was also consistent (CV = 1.12%), whereas greater variation was observed for $\sigma_{y}^{1}$, $\varepsilon_{y}^{1}$, and $\varepsilon_{y}^{2}$ (CV = 13.19%, 12.73%, and 12.87%, respectively). Nevertheless, all solutions produced similar force–displacement agreement, with MSE values ranging from 1.67 to 1.91 (mN)^2^.

The greater variability of the intermediate hardening point indicates that its exact location is less tightly constrained by the indentation response, although it provides additional flexibility in representing the nonlinear hardening behavior. Initial numerical testing showed that a bilinear hardening representation produced a substantially higher MSE for the same specimen (approximately 9.8 (mN)^2^ compared with 1.72 (mN)^2^ for the three-point representation), supporting inclusion of the intermediate control point. Variability in $\varepsilon_{y}^{2}$ had limited influence on the fitted response because the terminal stress $\sigma_{y}^{2}$ remained similar among the solutions. Overall, these results indicate good repeatability for the initial yield and terminal stress parameters, while the intermediate hardening parameters are less uniquely constrained by the indentation response.

The cumulative number of FEA simulations was not recorded for all optimization runs because only the converged optimization outputs were retained, and intermediate simulation files were not systematically archived due to storage limitations. However, for the representative cortical tangential case used in the repeatability analysis, approximately 750 FEA simulations were required per optimization run.

S2 Fig compares the experimental and optimized FEA force–displacement responses for representative cortical samples and shows close agreement throughout the indentation depth. The corresponding indentation stress–strain curves are also shown together with the optimized constitutive control points. While the initial yield point is informed by the experimentally derived response, the second and third control points are inverse-identified post-yield parameters and are not expected to coincide directly with the apparent experimental stress–strain curve. Beyond initial yielding, estimation of indentation stress and strain becomes increasingly uncertain because the evolving contact area is not directly measured and may be influenced by pile-up and sink-in. Therefore, the experimentally measured force–displacement response was used as the primary target for inverse optimization.

S1.3 μQCT-based RVE reconstruction and homogenization

μQCT imaging of the femur was performed in this study using the SkyScan 1276 CMOS system. A spatial resolution of 20 $\mu m$ was set for imaging which produced the final images with 2016 $\times$ 2016 pixels. Using this technique, RVEs from the femoral head and neck, the greater trochanter, the distal metaphysis, and the distal condyle were obtained in the form of DICOM data. The DICOM images were segmented using 3D Slicer [1]. These RVEs were extracted as stereolithography models from 3D Slicer and then post-processed in SpaceClaim [2] to avoid any geometrical errors such as self-intersections, open surfaces, and sharp edges.

The mathematical expressions for calculating the homogenized elasticity tensor are briefly discussed here, where the microscopic and macroscopic domains are denoted by y and Y, respectively. For a unit cell with a volume of $\left| Y \right|$, the effective elasticity tensor $E^{H}$ is given by:

$$\begin{aligned} E_{ijkl}^{H}=\frac{1}{\left| Y \right|}\int_{Y} E_{pqrs}\varepsilon_{pq}^{A\left( ij \right)}\varepsilon_{rs}^{A\left( kl \right)}dY \#\left( S1.8 \right) \end{aligned}$$

where $\varepsilon_{pq}^{A\left( ij \right)}= \varepsilon_{pq}^{0\left( ij \right)}-\varepsilon_{pq}^{*\left( ij \right)}$ and $\varepsilon_{rs}^{A\left( kl \right)}= \varepsilon_{rs}^{0\left( kl \right)}-\varepsilon_{rs}^{*\left( kl \right)}$. Here, $E_{pqrs}$ is the locally varying elasticity tensor, $\varepsilon_{pq}^{0\left( ij \right)}$ represent the test strains, and $\varepsilon_{pq}^{*\left( ij \right)}$ is the Y-periodic solution to the following boundary value problem:

$$\begin{aligned} E_{ijkl}^{H}=\frac{1}{\left| Y \right|}\int_{Y} E_{pqrs}\left( \varepsilon_{pq}^{0\left( ij \right)}-\varepsilon_{pq}^{*\left( ij \right)} \right)\left( \varepsilon_{rs}^{0\left( kl \right)}-\varepsilon_{rs}^{*\left( kl \right)} \right)dY\#\left( S1.9 \right) \end{aligned}$$

$$\begin{aligned} \int_{Y} E_{ijpq}\varepsilon_{pq}^{*\left( kl \right)}\frac{\partial\vartheta_{i}}{\partial y_{j}}dY=\int_{Y} E_{ijpq}\varepsilon_{pq}^{0\left( kl \right)}\frac{\partial\vartheta_{i}}{\partial y_{j}}dY \#\left( S1.10 \right) \end{aligned}$$

where $\vartheta$ is the Y-periodic displacement field.

S1.4 Variability of experimental and optimized material parameters

Table S4. CV results of trabecular and cortical bone in each region from the experimental results.

| **Region** | **Bone Type** | **Nanoindentation Experiments Parameters CV (%)** | | | | | | |
| --- | --- | --- | --- | --- | --- | --- | --- | --- |
|  |  | $\boldsymbol{E}$ | $\boldsymbol{\sigma}_{\boldsymbol{y}}^{\boldsymbol{0}}$ | $\boldsymbol{\varepsilon}_{\boldsymbol{y}}^{\boldsymbol{0}}$ | $\boldsymbol{\sigma}_{\boldsymbol{y}}^{\boldsymbol{1}}$ | $\boldsymbol{\varepsilon}_{\boldsymbol{y}}^{\boldsymbol{1}}$ | $\boldsymbol{\sigma}_{\boldsymbol{y}}^{\boldsymbol{2}}$ | $\boldsymbol{\varepsilon}_{\boldsymbol{y}}^{\boldsymbol{2}}$ |
| **FH** | Cortical | 12.3 | 18.4 | 10.7 | - | - | - | - |
|  | Trabecular | 5.6 | 5.2 | 13.6 | - | - | - | - |
| **PM** | Cortical | 8.3 | 10.1 | 5.2 | - | - | - | - |
| **CD** | Cortical | 16.0 | 18.0 | 5.4 | - | - | - | - |
|  | Cortical | 12.0 | 19.7 | 18.8 | - | - | - | - |
|  | Cortical | 10.5 | 15.8 | 10.3 | - | - | - | - |
| **DM** | Cortical | 14.9 | 18.2 | 17.2 | - | - | - | - |
|  | Trabecular | 18.2 | 23.3 | 6.0 | - | - | - | - |

Table S5. CV results of trabecular and cortical bone in each region from the optimization results.

| **Region** | **Bone Type** | **Optimization Parameters CV (%)** | | | | | | |
| --- | --- | --- | --- | --- | --- | --- | --- | --- |
|  |  | $\boldsymbol{E}$ | $\boldsymbol{\sigma}_{\boldsymbol{y}}^{\boldsymbol{0}}$ | $\boldsymbol{\varepsilon}_{\boldsymbol{y}}^{\boldsymbol{0}}$ | $\boldsymbol{\sigma}_{\boldsymbol{y}}^{\boldsymbol{1}}$ | $\boldsymbol{\varepsilon}_{\boldsymbol{y}}^{\boldsymbol{1}}$ | $\boldsymbol{\sigma}_{\boldsymbol{y}}^{\boldsymbol{2}}$ | $\boldsymbol{\varepsilon}_{\boldsymbol{y}}^{\boldsymbol{2}}$ |
| **FH** | Cortical | 15.2 | 19.2 | 10.6 | 12.5 | 28.5 | 12.5 | 15.8 |
|  | Trabecular | 6.7 | 19.9 | 23.9 | 24.2 | 30.2 | 14.9 | 4.4 |
| **PM** | Cortical | 6.9 | 11.2 | 9.0 | 23.3 | 7.1 | 11.4 | 16.2 |
| **CD** | Cortical | 3.3 | 8.6 | 5.9 | 11.9 | 8.5 | 5.1 | 22.4 |
|  | Cortical | 12.6 | 15.8 | 9.4 | 52.7 | 13.2 | 6.6 | 15.3 |
|  | Cortical | 8.1 | 18.1 | 17.8 | 21.6 | 43.2 | 13.6 | 28.9 |
| **DM** | Cortical | 12.5 | 19.6 | 14.7 | 13.2 | 20.8 | 16.9 | 20.4 |
|  | Trabecular | 21.1 | 24.8 | 4.8 | 11.9 | 13.9 | 10.3 | 25.8 |

S1.5 Homogenized orthotropic properties of trabecular RVEs

Table S6. Homogenization results for all RVEs. The moduli are reported in GPa.

|  | $\boldsymbol{RV}\boldsymbol{E}_{\boldsymbol{11}}$ | $\boldsymbol{RV}\boldsymbol{E}_{\boldsymbol{12}}$ | $\boldsymbol{RV}\boldsymbol{E}_{\boldsymbol{21}}$ | $\boldsymbol{RV}\boldsymbol{E}_{\boldsymbol{22}}$ | $\boldsymbol{RV}\boldsymbol{E}_{\boldsymbol{31}}$ | $\boldsymbol{RV}\boldsymbol{E}_{\boldsymbol{32}}$ | $\boldsymbol{RV}\boldsymbol{E}_{\boldsymbol{41}}$ | $\boldsymbol{RV}\boldsymbol{E}_{\boldsymbol{42}}$ | $\boldsymbol{RV}\boldsymbol{E}_{\boldsymbol{51}}$ | $\boldsymbol{RV}\boldsymbol{E}_{\boldsymbol{52}}$ |
| --- | --- | --- | --- | --- | --- | --- | --- | --- | --- | --- |
| $\boldsymbol{E}_{\boldsymbol{1}}$ | 1.53 | 2.35 | 1.12 | 0.67 | 0.60 | 0.43 | 4.24 | 1.12 | 0.71 | 0.35 |
| $\boldsymbol{E}_{\boldsymbol{2}}$ | 1.80 | 2.49 | 1.40 | 1.04 | 0.45 | 0.45 | 4.85 | 1.15 | 1.50 | 0.71 |
| $\boldsymbol{E}_{\boldsymbol{3}}$ | 2.43 | 2.98 | 1.83 | 1.08 | 1.08 | 1.00 | 5.62 | 2.70 | 1.26 | 0.36 |
| $\boldsymbol{G}_{\boldsymbol{23}}$ | 0.66 | 0.95 | 0.43 | 0.30 | 0.17 | 0.16 | 1.68 | 0.34 | 0.37 | 0.16 |
| $\boldsymbol{G}_{\boldsymbol{31}}$ | 0.83 | 1.05 | 0.60 | 0.42 | 0.25 | 0.27 | 2.00 | 0.60 | 0.54 | 0.20 |
| $\boldsymbol{G}_{\boldsymbol{12}}$ | 0.75 | 1.03 | 0.48 | 0.33 | 0.31 | 0.24 | 1.86 | 0.67 | 0.35 | 0.12 |
| $\boldsymbol{\nu}_{\boldsymbol{12}}$ | 0.24 | 0.25 | 0.20 | 0.16 | 0.25 | 0.22 | 0.24 | 0.24 | 0.13 | 0.11 |
| $\boldsymbol{\nu}_{\boldsymbol{13}}$ | 0.18 | 0.21 | 0.15 | 0.16 | 0.14 | 0.08 | 0.23 | 0.22 | 0.20 | 0.21 |
| $\boldsymbol{\nu}_{\boldsymbol{23}}$ | 0.20 | 0.22 | 0.18 | 0.20 | 0.12 | 0.15 | 0.25 | 0.24 | 0.21 | 0.28 |
| $\boldsymbol{\nu}_{\boldsymbol{21}}$ | 0.29 | 0.27 | 0.25 | 0.25 | 0.19 | 0.23 | 0.28 | 0.28 | 0.28 | 0.22 |
| $\boldsymbol{\nu}_{\boldsymbol{31}}$ | 0.28 | 0.27 | 0.24 | 0.25 | 0.25 | 0.20 | 0.29 | 0.30 | 0.35 | 0.22 |
| $\boldsymbol{\nu}_{\boldsymbol{32}}$ | 0.27 | 0.26 | 0.24 | 0.21 | 0.29 | 0.34 | 0.28 | 0.28 | 0.18 | 0.14 |

Here, $E_{1}$, $E_{2}$, and $E_{3}$ are the elastic moduli, $G_{23}$, $G_{31}$, and $G_{12}$ are the shear moduli, and $\nu_{12}$, $\nu_{13}$, $\nu_{21}$, $\nu_{23}$, $\nu_{31}$, and $\nu_{32}$ are the Poisson’s ratios in the three orthotropic principal planes.

References

1.	Fedorov A, Beichel R, Kalpathy-Cramer J, Finet J, Fillion-Robin J-C, Pujol S, et al. 3D Slicer as an image computing platform for the Quantitative Imaging Network. Magn Reson Imaging. 2012;30: 1323–1341. doi:10.1016/j.mri.2012.05.001

2.	ANSYS Inc. SpaceClaim. Canonsburg, PA, USA: ANSYS, Inc.; 2025. Available: https://www.ansys.com/products/3d-design/ansys-spaceclaim
